# Harnessing Protein-Ligand Interaction Fingerprints to Predict New Scaffolds of RIPK1 Inhibitors

**DOI:** 10.3390/molecules27154718

**Published:** 2022-07-23

**Authors:** Natália Aniceto, Vanda Marques, Joana D. Amaral, Patrícia A. Serra, Rui Moreira, Cecília M. P. Rodrigues, Rita C. Guedes

**Affiliations:** Department of Pharmaceutical Sciences and Medicines and Research Institute for Medicines (iMed.ULisboa), Faculdade de Farmácia, Universidade de Lisboa, 1649-003 Lisboa, Portugal; nataliaaniceto@ff.ulisboa.pt (N.A.); vismsmarques@ff.ulisboa.pt (V.M.); jamaral@ff.ulisboa.pt (J.D.A.); pfserra@ff.ulisboa.pt (P.A.S.); rmoreira@ff.ulisboa.pt (R.M.); cmprodrigues@ff.ulisboa.pt (C.M.P.R.)

**Keywords:** necroptosis, RIPK1, inhibitors, docking, machine learning, QSAR, virtual screening

## Abstract

Necroptosis has emerged as an exciting target in oncological, inflammatory, neurodegenerative, and autoimmune diseases, in addition to acute ischemic injuries. It is known to play a role in innate immune response, as well as in antiviral cellular response. Here we devised a concerted in silico and experimental framework to identify novel RIPK1 inhibitors, a key necroptosis factor. We propose the first in silico model for the prediction of new RIPK1 inhibitor scaffolds by combining docking and machine learning methodologies. Through the data analysis of patterns in docking results, we derived two rules, where rule #1 consisted of a four-residue signature filter, and rule #2 consisted of a six-residue similarity filter based on docking calculations. These were used in consensus with a machine learning QSAR model from data collated from ChEMBL, the literature, in patents, and from PubChem data. The models allowed for good prediction of actives of >90, 92, and 96.4% precision, respectively. As a proof-of-concept, we selected 50 compounds from the ChemBridge database, using a consensus of both molecular docking and machine learning methods, and tested them in a phenotypic necroptosis assay and a biochemical RIPK1 inhibition assay. A total of 7 of the 47 tested compounds demonstrated around 20–25% inhibition of RIPK1’s kinase activity but, more importantly, these compounds were discovered to occupy new areas of chemical space. Although no strong actives were found, they could be candidates for further optimization, particularly because they have new scaffolds. In conclusion, this screening method may prove valuable for future screening efforts as it allows for the exploration of new areas of the chemical space in a very fast and inexpensive manner, therefore providing efficient starting points amenable to further hit-optimization campaigns.

## 1. Introduction

Necroptosis is a form of programmed necrosis which has been explored for clinical application in cancer, inflammatory, neurodegenerative, and autoimmune diseases, in addition to acute ischemic injuries [1]. This is a non-apoptotic and caspase-independent cell death pathway triggered by death receptor ligands, tumor necrosis factor ligand superfamily member 6 (FasL), and tumor necrosis factor (TNFα), leading to a sequence of key events from transcription to stabilization and post-translational modifications of necrosome components, namely: (1) activation of tumor necrosis factor receptor 1 (TNFR1); (2) receptor interacting protein kinase-1 (RIPK1) ubiquitination by cellular inhibitor of apoptosis proteins (cIAPs) and phosphorylation by transforming growth factor-β-activated kinase 1 (TAK-1); (3) activation of receptor interacting protein kinase-3 (RIPK3) by RIPK1; (4) phosphorylation of mixed lineage kinase domain-like pseudokinase (MLKL) by RIPK3; and (5) assembly of an MLKL oligomer which translocates into the cell membrane, leading to cell lysis [2,3].

Inhibition of necroptosis as a therapeutic approach is very appealing because one of its key biological players, RIPK1, is a kinase that has an unusual allosteric binding site among kinases characterized by the so-called “DLG-out/Glu-out” inactive conformation [4]. This hallmark lends itself to highly specific molecular targeting. Following the discovery of the first specific inhibitor, necrostatin-1 (Nec-1) [5], multiple highly selective RIPK1 inhibitors were discovered through biological screening paired with structure-activity relationship (SAR) optimization, some of which were co-crystallized with their kinase target [4]. Among the molecules discovered, five have entered clinical trials for multiple conditions: GSK2982772 (Phase II, ulcerative colitis; Phase II, rheumatoid arthritis; Phase II, psoriasis), DNL747 (Phase I, Alzheimer’s disease), SAR443122 (Phase Ib, coronavirus disease 2019; Phase II, lupus erythematosus), SAR443820 (Phase I, multiple sclerosis), GSK3145095 (Phase II, pancreatic cancer), GFH312 (Phase I, inflammatory conditions) [6,7]. The uniqueness of the allosteric pocket is particularly desirable in targets such as kinases, as these are particularly prone to a high degree of promiscuity [8].

RIPK1 has proven to be a useful druggable target to modulate distinct disease processes [9]. However, so far, the discovery of RIPK1 inhibitors has typically relied on in vitro or small in silico screening where, at best, a focused set of compounds derived from already-known scaffolds is tested. Structure-activity relationship (SAR) studies resulting from such screening efforts are therefore not necessarily applicable to other new scaffolds. Gaining insight into the broader patterns that describe what enables a compound to bind to the allosteric back pocket of RIPK1 would therefore be extremely useful in guiding the optimization, design, and/or selection of new active compounds. Although many other kinases have been explored to develop in silico models using various structure- and ligand-based methods that predict inhibitors [10], only one study on QSAR modeling of RIPK1 inhibitors to find new SARS-CoV-2 therapies has been reported to date, where authors report no test performance for their classification model and low test performance (R^2^ < 0.35) for the regression model [11].

To bridge this gap, in this work we attempt to glean new information on the molecular determinants that drive RIPK1 inhibition by employing a synergistic approach of molecular docking and machine learning methods to a diverse set of active and inactive compounds. Analysis of the docking poses of different sets of RIPK1 inhibitors uncovered a *four-residue signature* that is statistically more frequent in inhibitors than in non-inhibitors. A consensus between this rule and quantitative SAR (QSAR) model predictions derived from machine learning was used to screen new inhibitor candidates from a diverse library of compounds. As a proof-of-concept of the usefulness of the knowledge gained from our in silico studies, we tested a small set of compounds using a phenotypic necroptosis assay and a biochemical (cell-free) assay with human RIPK1. We were able to identify seven compounds that are moderately active, which may be used as starting points to devise new families of RIPK1 inhibitors. This work indicates that our in silico screening protocol can be used as a fast and inexpensive approach to identify new diverse compounds that can successfully be employed as starting points for further development. This work is a pioneer attempt to propose predictive structural rules for RIPK1 inhibition.

## 2. Methods

### 2.1. Assembly of the Dataset of RIPK1 Inhibitors

We assembled a dataset of RIPK1 inhibitors by collating compounds and their experimental bioactivity data from ChEMBL 25 [12], a recent RIPK1 screen by Harris et al. [13] (termed “*the Harris dataset*” throughout), and a patent recently filed by Roche (termed “*the Roche dataset*” throughout) [14]. Compound structures from the RIPK1 inhibitors dataset were obtained either from the ChEMBL 25 SQL database (as SMILES), the raw data downloaded from PubChem BioAssays (as SMILES), or from their corresponding references (as 2D structure images or IUPAC names). Whenever IUPAC names were available, OPSIN [15] was used to obtain the corresponding SMILES. Whenever structures were only available as a 2D image, OSRA [16] was used to convert the images to SMILES.

ChEMBL data was accepted according to typical high-quality curation parameters, such as confidence score >7, bioactivity relation taking values of “=” or “>” (for activities above 2 μM) or “<” (for activities below 2 μM). Assays were required to be type “B” (for binding), and the organism was set as ”Homo sapiens.” As the aim was to build a classification model that distinguished between active and inactive compounds, all data were subsequently filtered by measurement type to include only *IC*_50_, *K*_i_, or *K*_d_. This type of aggregation is common for binary classification purposes [17,18].

The resulting activity readouts will be termed “bioactivities” throughout this work, and a compound was deemed active if it demonstrated bioactivity below 2 μM.

Canonical SMILES annotated in ChEMBL were washed and standardized using MOE2019.02 package [19] and MolVS 0.1.1 (https://molvs.readthedocs.io/en/latest (accessed on 14 July 2022)), where structures were converted into the dominant tautomer and protomer at pH = 7.4. The resulting structures were converted to InChIKeys using RDKit [20] to remove duplicates and each unique structure was assigned the minimum value among its duplicates.

**Figure 1:**
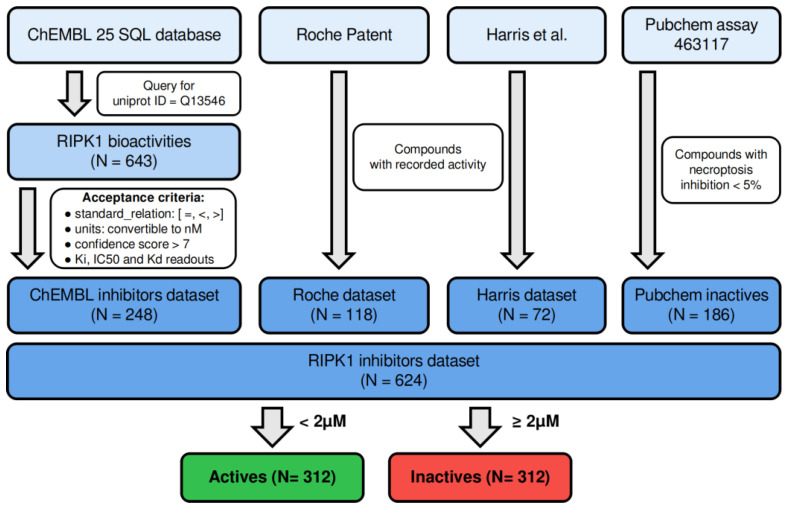
Overview of the data assembly workflow used to produce the dataset of RIPK1 inhibitors that was used in this work [13].

At the end of this workflow, a total of 248 datapoints were retained from ChEMBL. Additionally, to enrich the chemical space covered, inhibitory data was added to the ChEMBL data from Harris et al. [13], the “Harris dataset” (N = 72), and from a 2019 Roche patent [14], the “Roche dataset” (N = 118). The activity distribution of these three merged datasets is portrayed in Supporting Figure S1. The resulting bioactivity data was binarized, using an activity threshold of 2 μM, leading to a dataset composed of 71% actives and 29% inactives. The two classes were subsequently balanced by adding 186 inactives from a confirmatory necroptosis inhibition assay performed on L929 cells, available in PubChem BioAssays (AID:463117, pubchem.ncbi.nlm.nih.gov/bioassay/463117 (accessed on 14 July 2022)). These inactive compounds were randomly sampled from a total of 277 PubChem compounds indicating necroptosis inhibition <5%. This type of balancing with inactive data has been proven to improve machine learning performance [17]. The final RIPK1 inhibitors dataset was composed of 624 compounds. The complete data assembly workflow is summarized in Figure 1.

### 2.2. Preparation of Compound Structures for QSAR

All compounds from various datasets were subjected to molecular docking and machine learning/QSAR calculations, therefore, they underwent proper structure preparation prior to any calculations. All compounds were stripped of any salts and underwent structure standardization using MolVS 0.1.1 (https://molvs.readthedocs.io/en/latest (accessed on 14 July 2022)) and subsequent correction of protonation and tautomer states using MOE2019.02. Additionally, compounds were energy-minimized with Amber10:EHT in MOE2019.02 using a tolerance threshold of 0.01. Stereochemistry was kept unchanged throughout all compound preparation.

### 2.3. Molecular Descriptors, Morgan Fingerprints, Protein-Ligand Interaction Fingerprints (PLIFs), and PLIFs Similarity

The compounds were represented in the form of different types of molecular descriptors that can be divided into three different classes: (1) Physicochemical descriptors (N = 200), calculated from the standardized SMILES using RDKit; (2) Morgan fingerprints, calculated in RDKit, for a radius = 2 and 1024 bits; (3) Protein–ligand interaction fingerprints (PLIFs), calculated using PLIP [21] (python implementation), which exhaustively characterizes all possible residue–ligand contacts for a pose obtained with molecular docking. PLIP detects contacts based on atom type complementarity, distance, and angle, which were parameterized by the developers using experimental values. In the PLIF signature of a compound, for a residue contact to “exist,” at least four out of the top five poses must indicate some form of interaction with that residue, thus serving as a “robust” PLIF signature. This was used to avoid any bias of PLIFs towards the first pose and was prompted by the unpredictable relationship between the first pose’s docking score and activity (see results section).

We used PLIFs’ similarity for some analyses, which corresponds to the Tanimoto similarity between the ligand’s interaction fingerprint and that of the co-crystal ligand in the PDB being used for the docking calculations under analysis.

### 2.4. Molecular Docking Protocol Validation and Optimisation

All docking calculations were performed using GOLD Version 5.7.1 [22], in which the scoring function, number of scored poses, and protein structure were optimized. At the time this work was being developed, eight human RIPK1 X-ray structures of reasonable resolution (<3 Å) were available in the Protein Data Bank [23], namely, 4ITH, 4ITI, and 4ITJ [24], 4NEU [25], 5HX6 [26], 5TX5 [27], 6C3E, and 6C4D [28]. The structures were stripped of all molecules other than the protein and the co-crystal ligand (waters and ions) and were then aligned. Each PDB is a RIPK1 dimer where each chain is interacting with a ligand molecule and, since different chains are very similar and often possess alignment RMSDs below 0.5 Å, chain A was selected by default. Finally, protomer and tautomer states were corrected using the Protonate3D functionality to optimize the protein’s intramolecular hydrogen bond network. All preparation steps described above were performed in MOE2019.02.

The docking sphere set defined in the GOLD’s configuration file had a 10 Å radius and a center placed in ASP156’s nitrogen (a crucial residue for the interaction with ligands). First, a validation step was performed with all protein structures where each co-crystal ligand was subjected to self-docking against its original X-ray protein, and cross-docking against the remaining seven proteins. Then, an exhaustive search for the best match of scoring function (ChemPLP, GoldScore, ChemScore, and ASP) and X-ray structure (listed above) was performed for 1000 GA runs. Here, the best set of parameters is the one that best allows the reproduction of the experimental pose of the ligands (i.e., RMSD ≤ 2 Å between docked and experimental pose). After the best structure and scoring function pair were found, the calculations were re-run for 100 and 50 poses to optimize the exhaustiveness of pose exploration. The optimal parameters discovered (5HX6, GoldScore, 50 poses) were then applied to perform molecular docking on around 260 K compounds from the ChemBridge database (more details below).

**Figure 2:**
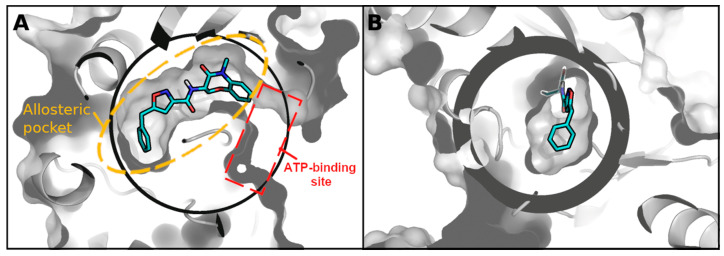
Binding sphere surrounding the binding pocket in 5HX6 (X-ray ligand indicated in cyan). Figure was produced using PyMOL and the sphere was calculated using the center and radius used in GOLD for all docking calculations. (**A**) Side view of the allosteric back pocket. The docking sphere also includes a portion of the ATP-binding site which begins immediately after the point where the ligand ends. (**B**) Bottom-up view of the allosteric back pocket.

Figure 2 portrays a cross-section of longitudinal view (A) and a view from the bottom of the pocket (B) of the docking sphere set in GOLD, surrounding the binding pocket and the X-ray ligand (cyan) of 5HX6. The region inside the sphere corresponds to the allosteric back pocket of RIPK1, which is “L” shaped and quite narrow, and is immediately adjacent to the ATP-binding pocket which, in Figure 2A, begins at the open end of the back pocket.

### 2.5. Development of a QSAR Model of RIPK1 Inhibition Using Machine Learning

**Figure 3:**
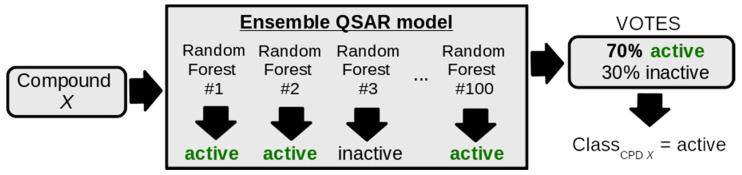
Example of how a prediction for each compound is obtained. Whenever a model was trained, the number of Random Forests in the ensemble was optimized.

To build a QSAR model able to predict RIPK1 inhibitors, we employed a random forest (RF) algorithm (scikit-learn [29]) trained on 70% of the RIPK1 inhibitors dataset (training set) and tested on the remaining 30% (test set) (random sampling). The data was annotated with three types of descriptors: 200 physicochemical descriptors from RDKit, 1024-bit Morgan fingerprints from RDKit and 6 key residue contacts previously selected from analysis of the ChEMBL data. Using the training set only, we optimized the number of trees (ranging between 100 and 1000, with a step = 100) and tested these against all possible combinations of feature subsets (physicochemical descriptors, Morgan fingerprints, and residue contacts). The best combination of several trees and feature set was selected according to the highest 10-fold cross-validation accuracy. The final model was then built as an ensemble of 100 RF models, trained with the optimal parameters previously selected. Each of the 100 RF models was trained with alternating random samples of 90% of the training set. The predicted class for each compound corresponds to the majority vote cast by the ensemble of random forest models, as exemplified in the scheme of Figure 3 and calculated as portrayed in Equation (1):

(1) $$majorityclass=\{if\frac{RFs\left(vote=active\right)}{100}\times 100>50\%:active;else:inactive\}$$

**Figure 4:**
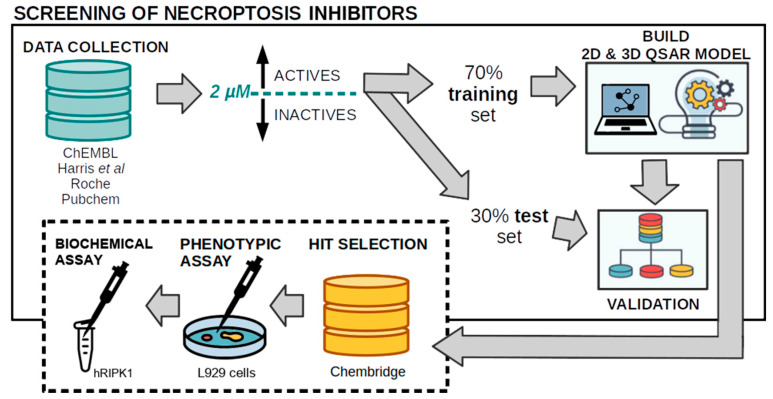
Workflow of the proposed methodology (computational and experimental) to predict RIPK1 inhibitors.

This RF ensemble was then applied to the test set to measure its ability to correctly predict new active compounds. Finally, as a proof-of-concept for its usefulness, we screened a large library of about 260 K commercial compounds from ChemBridge Corporation (more details below). The overall workflow described above is portrayed in Figure 4.

### 2.6. Virtual Screening and Compound Selection

The dataset of compounds to screen was gathered from the union of the EXPRESS-Pick and CORE libraries available from ChemBridge Corporation (~1.2 M unique compounds). All structures were downloaded from the vendor’s website in the SDF format, and were submitted to the compound preparation procedure. To allow for a more time-efficient screening (particularly in the case of docking), the dataset was filtered to keep compounds with a quantitative estimate of drug-likeness [30] (QED) > 0.85 and without PAINS substructures [31]. Both parameters were assessed using RDKit and these two filters produced a final dataset of 268,031 compounds which were screened through the docking and the QSAR platforms.

Hit compounds were selected from the overlap between the four-residue signature (LEU70, VAL75, ASP156, LEU157) and the PLIFs similarity >0.83 rule (details in the results section). PLIF similarity was calculated based on six key residues (LEU70, VAL75, LEU78, LEU129, ASP156, ASP157) selected for being robust interactions across the different PDB structures of RIPK1 (see discussion section). These 2 filters produced 1744 candidates, compounds that can simultaneously contact all 4 residues and indicate a similar binding mode (only considering the 6 key residues selected) to that of the co-crystal ligand in 5HX6. Candidates were selected for purchase using a consensus between the previous rules and the QSAR predictions (N = 66). To reduce this number to a set of compounds to test, as well as to prevent over-extrapolating from the known chemical space from which the 4-residue signature was derived, only the 50 compounds from the ChemBridge dataset with the largest common substructure to the Harris dataset were selected (named “in silico hits” set).

### 2.7. Phenotypic Assay of Necroptosis Inhibition

Fifty selected in silico hits were purchased and dissolved in dimethyl sulfoxide (DMSO, Sigma-Aldrich, St. Louis, MO, USA) for a final concentration of 100 μM after which three were excluded due to solubility issues. These were tested in a phenotypic assay of necroptosis inhibition using the L929 murine fibrosarcoma cell line (ATCC, Manassas, VA, USA). L929 cells were cultured in Dulbecco’s modified Eagle’s medium (DMEM), supplemented with 10% heat-inactivated fetal bovine serum and 1% antibiotic/antimycotic (all from Gibco, Thermo Fisher Scientific, Paisley, UK), and maintained at 37 °C under a humidified atmosphere of 5% CO_2_. L929 cells were seeded in 96-well plates at 0.5 × 10^4^ cellular density. After 24 h, cells were co-incubated with 10 ng/mL murine TNFα (Peprotech, London, UK) for necroptosis induction and 30 μM of selected hits for an additional 8 h. Nec-1 (Sigma-Aldrich) at 30 μM was used as a positive control for necroptosis inhibition. Adenylate kinase (AK) released from damaged cells was determined using the ToxiLight^TM^ Non-Destructive Cytotoxicity BioAssay Kit (Lonza, Basel, Switzerland) according to the manufacturer’s instructions. The luminescence signal was recorded using a GloMax^®^MultiDetection System (Promega, Madison, WI, USA). All measurements were performed in duplicate and all assays were performed in triplicate. AK readings were used as a cell death readout and the results were reported as fold-to-control (DMSO) to normalize for variability across different plates.

### 2.8. Biochemical Assay of RIPK1 Inhibition

RIPK1 activity was tested upon treatment with each compound using a radiometric ATP-competitive assay through the KinaseProfiler™ service provided by Eurofins Discovery Services (assay 16-022KP). Briefly, the compounds were prepared in 100% DMSO and added to a final reaction volume of 25 μL containing human RIPK1 (hRIPK1), 8 mM MOPS (pH 7.0), 0.2 mM EDTA, 100 μM KKRNRTLTV, 10 mM Magnesium acetate, and [γ-33P-ATP] (concentration used according to RIPK1’s *K*_m_). This mixture was incubated for 40 min at room temperature, after which 5 μL of a 3% phosphoric acid solution was added to stop the reaction. Then, 10 μL were collected from the reaction medium and spotted onto a P30 filtermat, washed three times for 5 min with a solution of 75 mM phosphoric acid once in methanol, followed by drying and scintillation counting.

### 2.9. Performance Evaluation

Prediction performance was measured using the precision for active compounds and overall accuracy, which were calculated using Equations (2) and (3), portrayed below:

(2) $$Precision=\frac{True\;Actives}{Predicted\;Actives}$$

(3) $$Accuracy=\frac{True\;Actives+True\;Inactives}{Predicted\;Actives+Predicted\;Inactives}$$

Additionally, we used enrichment metrics such as the ratio of actives (inhibitors) over inactives (non-inhibitors) or % of enrichment for actives (truly predicted actives/predicted actives) × 100.

### 2.10. Applicability Domain

To minimize the acceptance of over-extrapolated predictions, i.e., predictions that are outside the applicability domain of the machine learning model, an adaptation of the reliability-density neighborhood (RDN) metric [32] was used to control the accepted prediction produced by the QSAR model. Similarly, over-extrapolation produced by the docking model was controlled by enforcing that a sufficiently large common substructure was shared with any compound in the Harris dataset. This was measured as the number of heavy atoms of the largest maximum common substructure between a query compound and each compound in the Harris dataset. This applicability domain filter for docking resulted from preliminary experiments where we observed that using the contacts signature selected many inactive compounds from a proprietary set of compounds, with small common substructures shared with the RIPK1 inhibitors dataset.

### 2.11. Analysis of Chemical Novelty for In Silico Hit Compounds

We determined the maximum common substructure (MCS) (or largest common scaffold) between each in silico hit compound and all compounds in the full RIPK1 dataset (ChEMBL, Roche, Harris, and PubChem datasets). We then gathered the pairs of hit + train compounds that shared the largest scaffold and plotted the ratio of shared/non-shared heavy atoms in every pair.

### 2.12. Data Analysis and Visualisation

The machine learning step was performed using Scikit-learn 0.23.2 and raw data handling and analysis were done using pandas 0.24.1, NumPy 1.15.4, and SciPy 1.2.1. Handling of chemical structures, calculation of molecular descriptors and Morgan fingerprints, maximum common substructure analysis, R-group decomposition, and production of 2D images were performed using RDKit 3 March 2020. Dunnett’s test, designed for multiple comparisons against a control, was implemented in Python 3.7.4 as described by Kanji [33]. This test was applied to analyse the significance of the observed differences between compounds and the TNFα control in the phenotypic assay. A *p*-value < 0.05 was considered significant. Three-dimensional images and analysis of pose positions were produced using PyMOL v1.8.4.0 [34]. A comparison of the different binding sites was performed using the Electron Density Support for Individual Atoms (EDIA) module [35] and the SIENA module [36] in the ProteinsPlus web server [37].

The graphical representation of the chemical space was performed by running all compounds through scikit-learn’s t-SNE [38] function, applied to 1024-bit Morgan fingerprints. This effectively compresses the original 1024 dimensions to two dimensions where, in theory, compounds retain relative proximity to each other, similar to the 1024-dimensions space.

## 3. Results and Discussion

### 3.1. Chemical Space Analysis of RIPK1 Inhibitors

**Figure 5:**
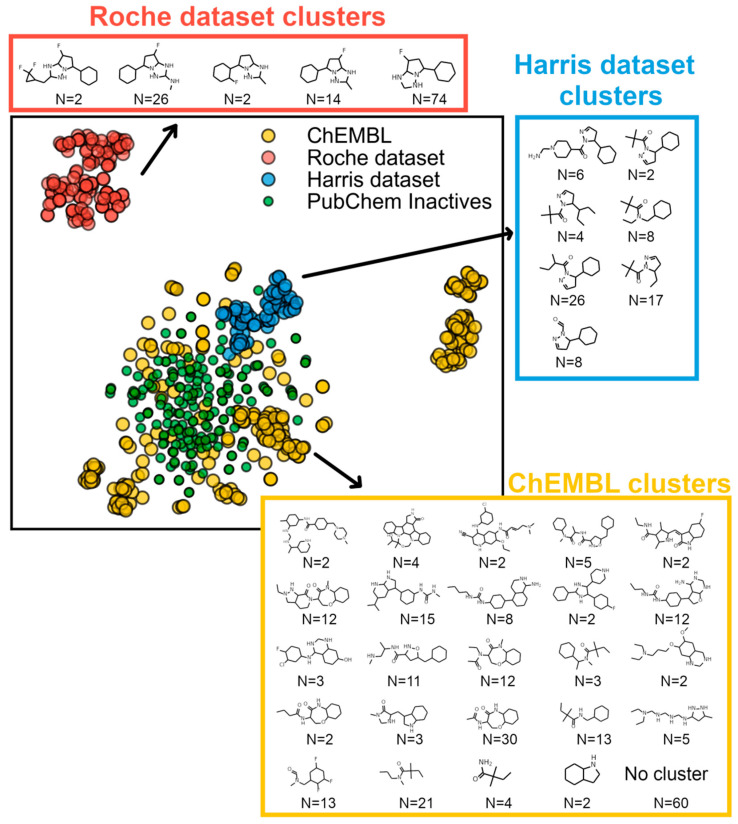
Chemical space distribution of RIPK1 inhibitors, produced using t-SNE over 1024-bit Morgan fingerprints. Relevant scaffold structures from each dataset were identified by hierarchical clustering, followed by maximum common substructure (MCS) decomposition.

At the beginning of this project, both chemical structures and bioactivity data of RIPK1 inhibitors were collected from ChEMBL (2008–2018). However, we later realized that inactives were under-represented (40%) and that only a relatively small amount of data (N = 248) fulfilled typical quality criteria (see details in the methods section). To overcome this issue, and to allow for the widest coverage in chemical space possible when training a machine learning QSAR model, we manually extracted additional data from two additional public sources, which yielded two datasets each—the Harris dataset and the Roche dataset (see methods section for details). This yielded a considerable expansion in chemical space coverage, which originally corresponded to just the yellow points, as portrayed in Figure 5. To inspect the chemical diversity observed by the distribution of points in Figure 5, we clustered compounds and gathered each cluster’s largest common scaffold (i.e., its MCS). All clusters are identified with their corresponding scaffold in Figure 5. This confirmed that new scaffolds were added with the new data collected. High chemical diversity was also detected through the occurrence of a large portion of compounds with no cluster affiliation (N = 60) (Figure 5). This complete dataset was used subsequently to build a QSAR model. A molecular docking-based screening model (i.e., interaction rules) was also built but using ChEMBL data alone, which occurred prior to new data being gathered. Thus, we used the additional datasets to test the prediction rules previously derived.

### 3.2. Validation of Molecular Docking Calculations

After the data was collected and analyzed, we sought to build a predictive molecular docking model. Prior to any docking calculation, we optimized the best combination of protein structure and scoring function, which entailed testing eight crystal structures available at the time experiments were first started (listed in the methods section) against four scoring functions implemented in GOLD. No protein-scoring function pair was able to reproduce all the ligands’ experimental pose (i.e., yield a docked pose with RMSD ≤ 2 Å of the experimentally determined pose in cross-docking validation). Supporting Figure S2. A indicates that GoldScore reproduced the largest number of experimental poses in 6 PDB structures (4ITH, 4ITI, 4NEU, 5HX6, 5TX5, and 6C3E), followed by ChemPLP which did so for 5 PDB structures. After selecting GoldScore, cross-docking calculations for 1000 runs indicate that structure 5TX5 reproduced the largest number of co-crystal ligands (Supporting Figure S2B) however, re-running docking for exhaustiveness of 50 runs indicates 5TX5 among the structures with high variance between the top poses achieved (Supporting Figure S3). This is an indication that 5TX5 might lack robustness in the poses produced for any given screened compound. 5HX6, conversely, indicated the robustness of all poses produced in cross-docking regardless of exhaustiveness level. Therefore, the GoldScore fitness function paired with 5HX6 as reference structure were the selected conditions to run all docking calculations. Search exhaustiveness of 50 runs was used instead of 1000 as it produced results comparable to 1000 poses (Supporting Figure S4) and allows for a viable calculation time for large datasets.

The inability to reproduce the experimental pose of all ligands appears to be due to the varying spatial constraints of the different binding pockets across structures, as calculated with EDIA (Supporting Table S1), which indicates that 4ITH, 4ITI, and 4ITJ have a less elongated pocket (less “tube-like”), whereas 5HX6, 5TX5, 6C3E, and 6C4D have longer pockets, evident by a smaller ellipsoid b/a ratio for the second group. 4NEU also appears to demonstrate an approximately tubular shape, similar to the second group. However, running SIENA with the “ligand pose comparison” option on the ProteinsPlus webserver indicates that, regardless of the PDB structure used as a query, 4NEU is never included as possessing a similar binding site to other RIPK1 PDB structures. This implies that 4NEU has a very different binding site from all other PDB structures. As a result, it would be very challenging for anyone to reproduce all co-crystal ligands included in this study. Despite the issue of the variable size/shape of RIPK1’s allosteric back pocket, 5HX6 demonstrates the ability to robustly reproduce the pose of both larger (5HX6, 5TX5, and 6C4D) and smaller (6C3E) ligands (different docking trials produce similar RMSD).

### 3.3. Docking Score and Ligand Efficiency Do Not Correlate with Activity

Using the parameters previously optimized, we first performed a molecular docking run for ChEMBL dataset to establish if docking score (or ligand efficiency, LE) would be good predictor of RIPK1 inhibitors. As we envisioned screening a large commercial library at the end of the project, we used 50 poses to build all predictive models and rules derived from docking data, after ensuring that the scores obtained using 1000 and 50 poses were sufficiently similar for the ChEMBL dataset (Supporting Figure S4).

**Figure 6:**
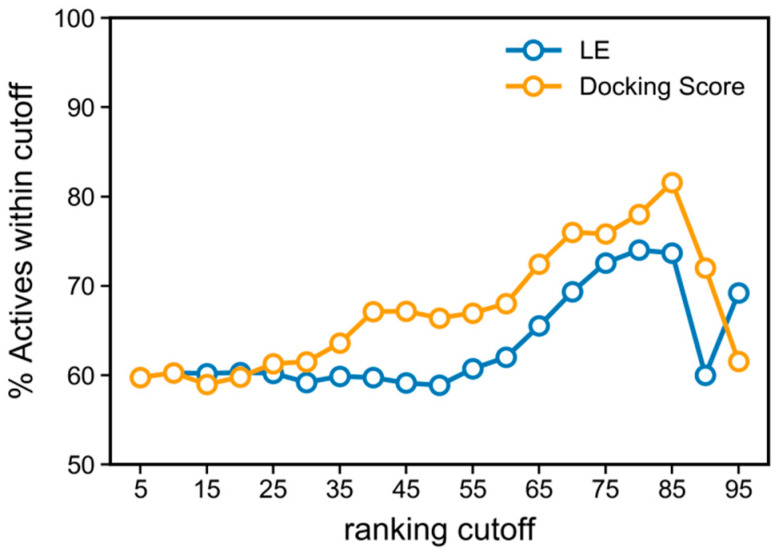
Enrichment curve with docking score and LE, applied to the ChEMBL dataset.

After docking the ChEMBL dataset, the results obtained clearly prove that the score alone does not indicate a reliable correlation with bioactivity (Figure 6), observed by a drop at the right end of the enrichment curve, which implies that the highest 15% docking scores or LE values portray a steady increase in inactives. A good curve is characterized by a steady increase or maintenance of the percentage of actives retrieved, as the selection cut-off becomes stricter, therefore enriching the selected compounds with actives.

The enrichment drops at the top of the docking scores rank, likely as a result of the well-known limitations associated with docking scores which, when used alone, are prone to rank compounds in a manner that does not correlate with activity or affinity [39,40]. Rather, docking excels in the quality of docking poses produced hence we decided to build a screening model based on the protein-ligand interactions that are associated with these poses.

The decision to not directly use docking scores or ligand efficiency as a parameter to select new compounds was later validated by results obtained with a new dataset consisting of an aggregate of the PubChem (inactives), the Harris, and the Roche datasets (Supporting Figure S5A). The results from these new data indicated that both docking outputs place inactives in the same distribution as actives, with inactives occupying slightly higher values in both parameters.

Additionally, comparing the enrichment curve produced by docking and LE obtained with 5HX6 versus using a more spherical pocket (i.e., 4ITH) confirms the initial hypothesis that 5HX6 would allow larger coverage across different ligands, leading to a better separation between active and inactive compounds (See Supporting Figure S5B).

### 3.4. PLIFs Similarity as a New Guideline to Find RIPK1 Inhibitors

**Figure 7:**
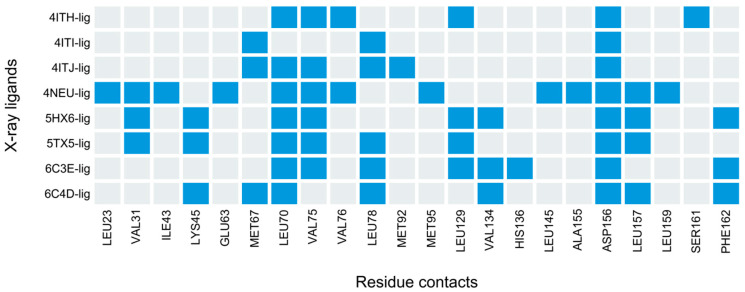
PLIF signatures for all X-ray ligands, where blue indicates the presence of contact of any kind between a residue and each ligand, and grey indicates the absence of interaction.

**Figure 8:**
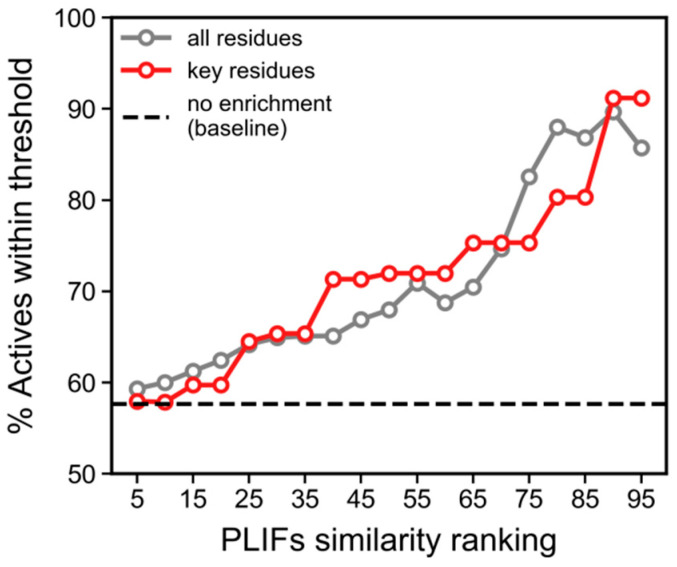
Enrichment curves for ChEMBL compounds, produced using PLIF similarities calculated from docking contacts with RIPK1 (5HX6, 50 poses). PLIF similarities were calculated with all residue contacts (N = 81 residues) portrayed by the grey curve, versus the six key residues that indicated frequent interactions among all crystal structures (i.e., LEU70, VAL75, LEU78, LEU129, ASP156, ASP157), portrayed by the red curve. Larger similarity ranking values on the X-axis corresponds to higher similarity.

The issue of lack of correlation between docking scores (or LE) and activity, which led to a poor enrichment towards higher values of docking scores, prompted us to use an alternative approach to harness the information produced by the docking calculations. To do so, we calculated the protein–ligand interaction fingerprints (PLIFs), and used these to quantify the PLIFs similarity of binding modes between each ChEMBL compound and the reference X-ray ligand (portrayed in Figure 7, under “5HX6-lig”). This metric produced a drastic improvement from the previous enrichment curve, as demonstrated by the grey curve in Figure 8 (corresponding to PLIFs obtained with 5HX6), with enrichment of actives reaching just below 90% (vs < 74% for LE). The PLIF similarity function also allowed us to correct for compound size (following the same rationale for the correction applied to docking scores, LE). A compound that shares all the interactions of the reference ligand by virtue of simply being large would be penalized, as it also indicates additional contacts not portrayed by the reference ligand.

In an attempt to further improve the enrichment rate of the “all residues” curve in Figure 8 (grey curve), we attempted to reduce the number of residues considered in the PLIF similarity measurements, as some of the contacts might be solely a result of particular characteristics of the structure of the reference ligand, and thus not well generalizable to other compounds. To do so, we kept only the residues that interact with at least half of the crystal structures (Figure 7), which resulted in the selection of six residues: LEU70 (seven ligands), VAL75 (six ligands), and LEU78 (five ligands) which belongs to the binding loop, LEU129 (five ligands) which belongs to the αE helix, and finally ASP156 (eight ligands) and LEU157 (N ligands) which are the catalytic residues belonging to the DLG/DFG motif found in the activation loop. LEU157 also participates in the regulatory spine and regulates the access to the active site pocket [13]. Recalculating the similarity using exclusively these six residues slightly improved the enrichment curve (Figure 8, red curve). From this analysis, we concluded that PLIF similarity ranking >80 (i.e., 80th percentile) would be a good cut-off to select inhibitors, as it allowed for covering the largest number of compounds with sufficiently high enrichment (~80%) (this percentile cutoff corresponds to similarities above 83%).

Testing the use of the six-residue PLIFs similarity on new datasets (Harris, Roche, and PubChem inactives) indicated that the previously selected cut-off (>83% similarity) only allows selecting three compounds from the test data. Such low data coverage means that these additional datasets cannot truly test the PLIF similarity cut-off derived from ChEMBL, which is likely because the external datasets have been intentionally created to populate a novel chemical space. However, the Harris and Roche datasets demonstrate 92% enrichment for actives at their own 80th percentile cut-off (which corresponds to 60% PLIFs similarity), which indicates that PLIF similarity also correlates with activity in additional datasets beyond ChEMBL. Surprisingly, when PubChem inactives were added to the two previous datasets, the enrichment rate dropped to 38%, which indicates that inactives overlap actives from Harris and Roche datasets with respect to PLIF similarities.

From all the results in this section, we conclude that PLIFs similarity can be used as an indicator of activity, as it produced high enrichment among the Roche and Harris datasets as well as with ChEMBL data, but it cannot be used as a sufficient condition for activity due to the simultaneous enrichment of PubChem inactives. Therefore, we sought to find an additional criterion that could be used as a confirmatory tool to predict new actives.

### 3.5. Deriving a New 4-Residue Rule to Predict RIPK1 Inhibitors

**Figure 9:**
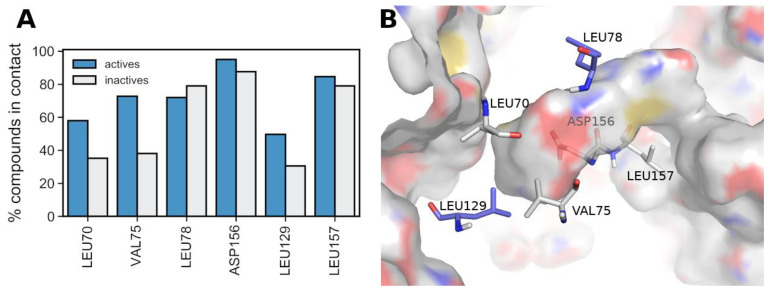
(**A**) Frequency (%) of contacts between the six key residues involved in binding with active versus inactive compounds. (**B**) The six key residue contacts selected from common shared contacts across RIPK1 X-ray structures. The four residues in grey correspond to the signature of simultaneous residues that, as a group, are statistically more prevalent in actives than in inactives across the multiple datasets.

To derive an interaction signature that is predictive of RIPK1 inhibition we performed an exhaustive analysis of the key residue contacts (LEU70, VAL75, LEU78, LEU129, ASP156, ASP157), using ChEMBL data alone. Observing each of the six residues individually does not indicate a particularly good separation between actives and inactives (Figure 9A). However, when higher complexity contact patterns were considered (i.e., all possible combinations of 2, 3, 4, 5, and 6 residues, composing 57 combinations in total) it was revealed that the 4-residue combination [LEU70, VAL75, ASP156, LEU157] was linked with the largest difference of contacts in actives *versus* inactives (2.5 times more frequent in actives than in inactives), and also accompanied by a significant coverage of actives (61%), as portrayed in Supporting Figure S6.

All four residues simultaneously interact with 61% of actives but only with 25% of inactives. This is aligned with the experimental knowledge gained from the different X-ray structures, which indicates that, in order to successfully inhibit RIPK1, a ligand must stabilize the protein in its inactive conformation by engaging multiple residues from opposite sides of the allosteric back pocket simultaneously [24]. Therefore, it is reasonable that multiple-residue engagement offers better differentiation between actives and inactives, compared to observing single residue contacts. The original six residues selected earlier are portrayed in Figure 9 (blue and grey residues), including the four residues associated with actives (indicated in grey), and essentially suggest that multiple contacts with residues all around the pocket are associated with the ability to inhibit RIPK1 (additional perspectives of the pocket surrounded by these key residues is portrayed in Supporting Figure S7).

**Figure 10:**
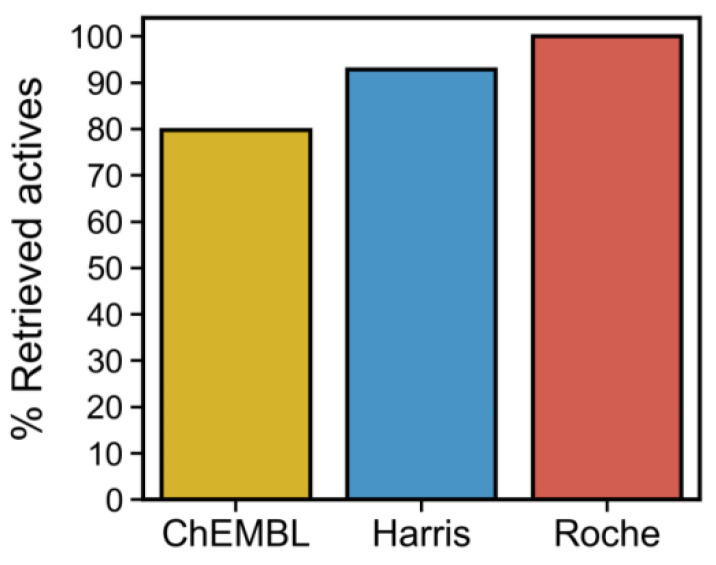
Performance of the 4-residue contact rule (LEU70, VAL75, ASP156, and LEU157) to filter actives from inactives from RIPK1 inhibitors datasets. The simultaneous occurrence of these four contacts is an adequate predictor of inhibition across the different subsets considered in this work (ChEMBL, Harris, and Roche subsets).

To assess whether the ability to predict actives can be extended to additional data, we tested this four-residue rule on the Harris and Roche datasets which cover different scaffolds than those in ChEMBL dataset. Figure 10 indicates that this structural signature can successfully select actives.

Evaluating this signature of residue contacts in all inactives (N = 632) in the PubChem phenotypic screen from which our inactives were drawn indicates that only 27% of compounds establish contacts with all four residues simultaneously. This is additional evidence in support of the value of this signature to predict binding leading to both inhibition and induction of RIPK1’s activity.

From these analyses, we concluded that this four-residue signature was a robust rule to predict RIPK1 actives, and it was used as a required condition to predict actives among the ChemBridge library, alongside the PLIF similarity filter (which will be treated as a non-essential pre-requisite).

### 3.6. Building a Machine Learning Model to Predict Necroptosis Modulators

**Figure 11:**
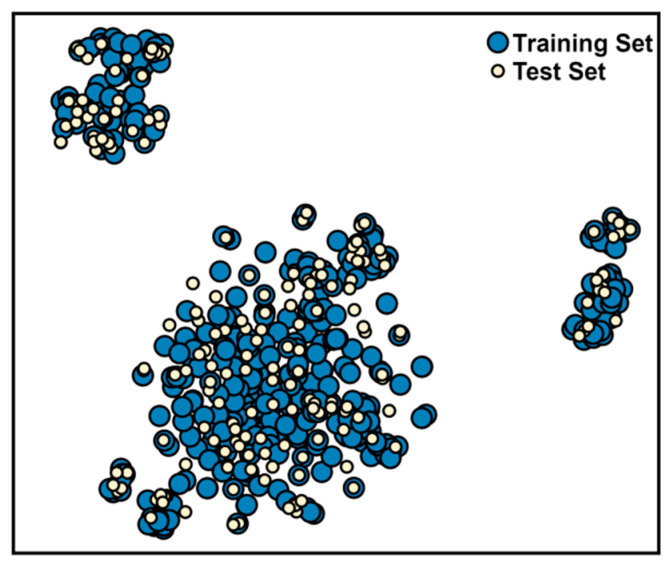
Chemical space distribution of training (blue) and test (cream) sets of the complete RIPK1 inhibitors dataset.

Next, we focused on building a machine learning model that could provide a ligand-based perspective to complement the two previous molecular docking-derived predictive rules. The RIPK1 inhibition dataset (aggregate of all subsets, namely, ChEMBL, Harris, Roche actives, and Pubchem inactives datasets), composed of 312 actives and 312 inactives, was split into a training (70%) and a test set (30% of data). This split was kept fixed for all models assessed in this work, by using the same random seed throughout. The training set was used for modeling and model optimization, and the test set was reserved for the final performance assessment. We ensured that the chemical space of the training and test sets had comparable coverage (Figure 11), given that we want to maximize the chemical space learned in training.

After hyperparameter optimisation the best 10-fold cross-validation accuracy (89.9 ± 2.7%) was obtained when using a random forest of 700 trees, trained on molecular descriptors and residue contacts. We provided the six key residues previously uncovered as the residue contact descriptors, rather than providing the four-residue signature derived from exploring trends in the six key residues. This ensures that the machine learning model is free to derive its own trends from the data. This accuracy level indicates that the establishment of a structure-activity relationship for RIPK1 inhibition was possible. These results led to the selection of molecular descriptors and residue contacts as the optimal feature, set to build the final QSAR classification model. The selection of the model architecture relying on cross-validation performance is essential as it avoids overfitting (overinflated prediction performance against the test set and potential loss of performance against new data).

**Figure 12:**
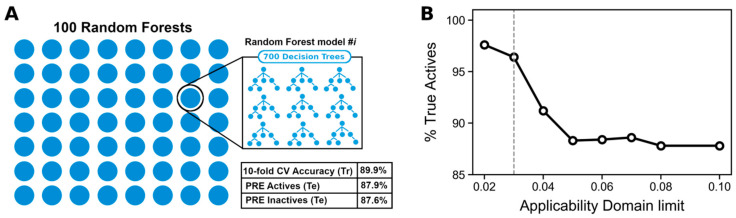
(**A**) Conceptual representation of the QSAR model, which consists of an ensemble of 100 random forests where each random forest model is composed of 700 decision trees. The table in the lower corner summarizes training and test performance. Tr: Training set, Te: Test set. (**B**) Applicability domain curve of the final QSAR model, where % True Actives refers to the test set.

Finally, we built an ensemble of 100 random forest models using the parameters previously optimized, as using an ensemble allows for more robust predictions (model structure represented in Figure 12A). This model produced a precision for actives and inactives of 87.9 and 87.6%, respectively, using no additional applicability domain filter. Employing the RDN applicability domain method enabled even higher precision values when more stringent limits were enforced (Figure 12B). However, the cost of using a stricter cut-off is fewer “accepted” predictions (i.e., predictions accepted within any given applicability domain cut-off). We opted for using a maximum applicability domain limit of 0.03 (dashed line in Figure 12B) as this allows for a good compromise between a low error rate among predicted actives (96.4% precision) and enough coverage (30% of test compounds). Overall, this filter controls which predictions are accepted, considerably decreasing the false positive rate, and has led to a precision of close to 100% (the rate at which predicted and accepted inhibitors are actual inhibitors).

The most important descriptors in our QSAR classification model were related to steric and electronic effects surrounding each atom in every molecule (MaxAbsEStateIndexm, MaxEStateIndex, MinEStateIndex), van der Waals surface areas of low partial charge (PEOE_VSA6), and non-polar atoms (SlogP_VSA5).

The six residue contact descriptors were scored at around 50% of the importance ranking within the QSAR model, which is not surprising given that the four-residue or the six-residue PLIFs similarity is not applicable to all actives, but rather they work to distinguish the actives they do cover from any inactives. Interestingly, LEU70 was placed at the top 30% of the importance ranking in 1 of the 100 random forests in the ensemble.

**Figure 13:**
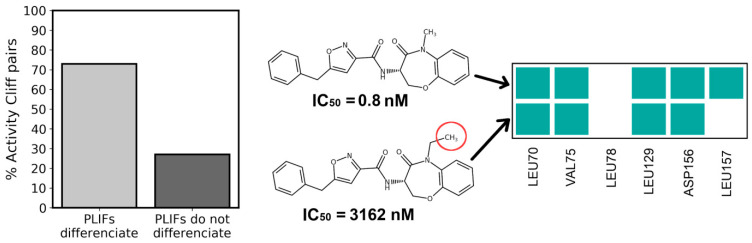
Six-residue PLIFs differentiate most of the activity cliffs in the RIPK1 inhibitors dataset. An example of an activity cliff present in the RIPK1 inhibitors dataset is displayed, where two compounds vary by a single methyl group, yet indicate a drastic difference in activity.

Furthermore, docking results provided useful tridimensionality to the QSAR model. A very common issue in QSAR modelling is the inability to set apart very similar structures which have very different activities—the so-called “activity cliffs.” This is caused by the fact that similar structures have largely the same 2D features. In this work, we observed that PLIF similarity was able to differentiate activity cliffs (Sim > 0.65) that drastically differ in activity (>0.5 μM of difference) (Figure 13).

In an even more extreme case, upon analyzing the contact signature of two activity cliff isomers reported by Harris et al. [13], the active S-enantiomer indicated three contacts out of the six key residues, while the inactive R-enantiomer indicated only one. Among all descriptors used in this work, only PLIFs allow for differentiating the last pair of activity cliff compounds, because all 2D features (physicochemical and Morgan fingerprints) are precisely the same between the two compounds and the docking scores are virtually the same (49.22 and 49.44) between them.

### 3.7. Virtual Screening of the ChemBridge Library Using the Docking-Based (4-Residue Signature and the PLIFs Similarity Filter) and the QSAR Models

**Figure 14:**
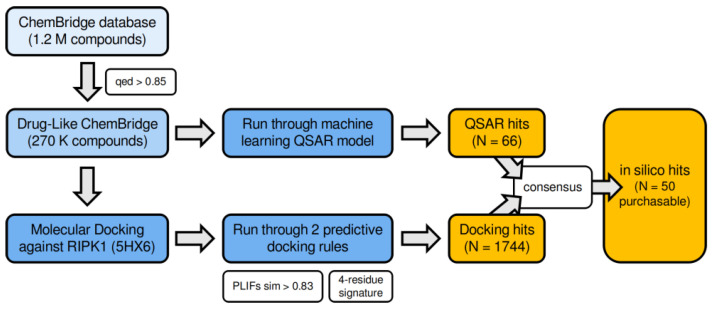
Summary of the screening workflow applied to the ChemBridge library of compounds to obtain the 50 in silico hits purchased for testing.

Once the final model was built, a drug-like subset of ~270 K compounds from ChemBridge (see methods section) was screened through the docking filter and through the QSAR classification model (see the summary scheme in Figure 14). From this subset, 1744 compounds indicated the 4-residue interaction signature and a PLIF similarity to 5HX6’s ligand of at least 0.83, meaning they would be expected to fit nicely inside RIPK1’s allosteric back pocket. A summary of the distribution of all ChemBridge drug-like candidates with respect to the two docking-based rules is demonstrated in Supporting Figure S8.

**Figure 15:**
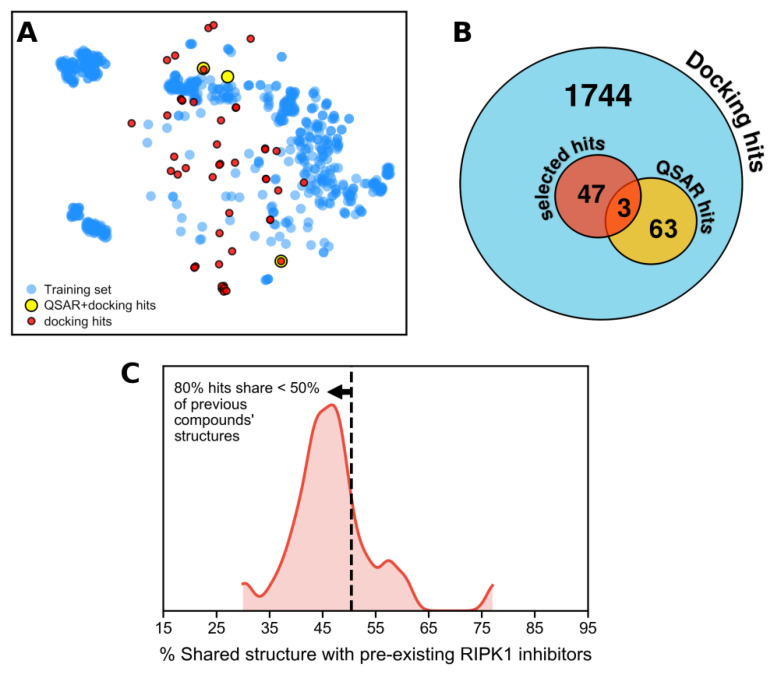
(**A**) Distribution of the 50 in silico hits (yellow and red) with respect to the RIPK1 inhibitors dataset (blue). (**B**) Overlap of subsets of in silico hits obtained, resulting from Docking predictions and machine learning QSAR predictions. (**C**) Quantifying chemical novelty through the distribution of the percentage shared structure between the in silico hits and their closest compound in the RIPK1 inhibitors dataset.

**Figure 16:**
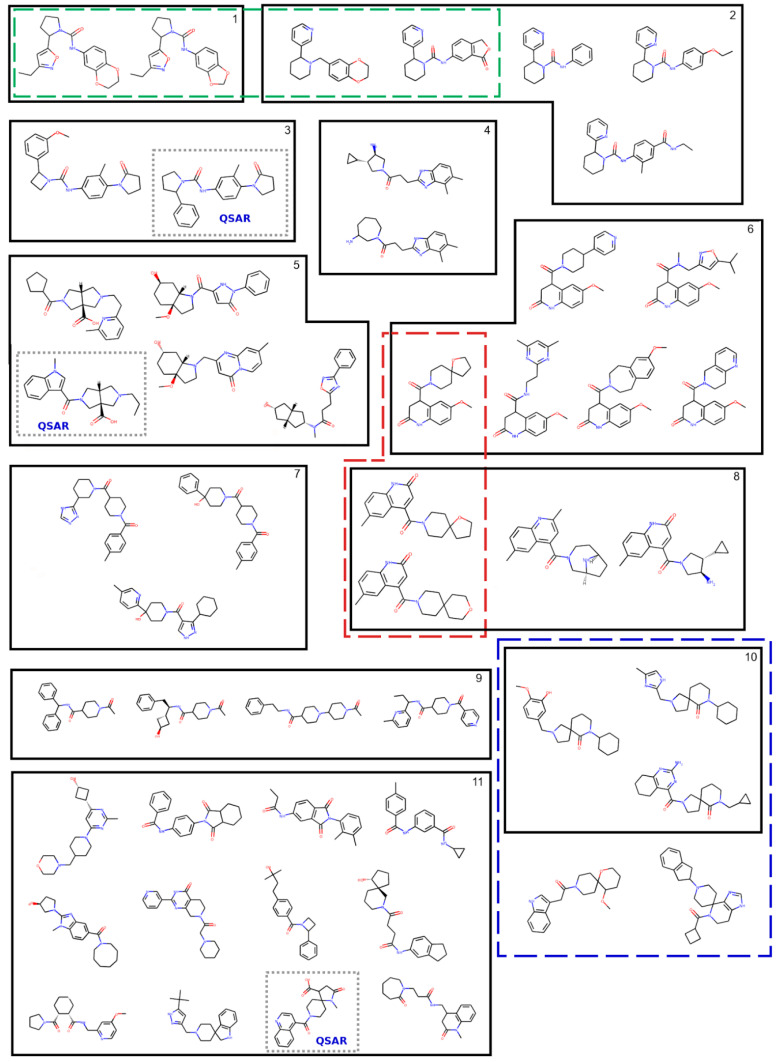
In silico hits selected for in vitro testing (N = 50). Compounds are clustered with hierarchical clustering applied to Morgan fingerprints. Clusters are generally ordered from top to bottom according to descending inter-cluster median similarity. Cluster 11 should be regarded as the pooled compounds that indicate generally low similarity to the remainder of the compounds. Molecules were aligned by common substructure using the “GenerateDepictionMatching2DStructure” functionality in RDKit, to aid visual comparison of molecules.

Among these docking hits, 66 compounds coincided with the reliably predicted hits resulting from the QSAR model. Since many of these were not readily available in the required quantity (5 mg) for biological evaluation (or were not available at all), we populated the in silico hits list with additional docking hits, for a total of 50 compounds (i.e., in silico hits set) (Figure 15B). This was the final set of purchased compounds, and it can be found in Figure 16 and Supporting Figures S9–S11.

The fact that only 66 compounds were predicted to be active among ca. 200,000 initial compounds is an indication of how different the training set chemical space from the ChemBridge database is. However, it should be noted that both sets of predictions were controlled with their own applicability domain filter to prevent over extrapolation. The complete overlap from QSAR and docking hits indicates a high level of agreement between the more general QSAR model which captures 2D molecular patterns related to RIPK1 inhibition, with the two rules derived from the docking model, which addresses the 3D complementarity to RIPK1’s allosteric back pocket. This is particularly relevant since, beyond the efforts to elucidate the SAR of RIPK1 modulation in chemical series, to our knowledge no attempts have been made to leverage public bioactivity data on necroptosis or RIPK1 modulation toward a broader QSAR predictive model.

In the same line as our work, a recent work by Rodríguez-Pérez and Bajorath [41] also used PLIFs for the development of a machine learning model that predicts binding modes among kinases with high accuracy, but included only X-ray data. Many other prediction models using molecular docking, QSAR modeling, pharmacophore modeling, and molecular dynamics have been reported for different kinases, as summarized in a recent review by Gagic et al. [10]. Particularly, Cruz et al. used QSAR, pharmacophore-based virtual screening, and molecular dynamics to design new RIPK2 inhibitors [42]. To our knowledge, only a single work has been published on QSAR modelling of RIPK1 where the authors modelled a variety of SARS-CoV-2 targets to discover new therapies for COVID-19. In this work, they modelled RIPK1 inhibitors from ChEMBL, but it is not clear how many compounds were used. The authors built both classification and regression models but reported no test set performance for the former and a low R^2^ for test data with the latter (best model = 0.35) [11]. The lack of more comprehensive modelling efforts focused on RIPK1 inhibition for drug discovery, paired with the fact that many other kinases have proven to be modellable, supported our goal to build a predictive in silico model to predict RIPK1 inhibition.

The chemical space placement of the 50 candidates with respect to the known chemical space of RIPK1 actives and inactives is demonstrated in Figure 15A (note that distances in this plot are not at scale from real distances, but rather define relative proximity). It is evident that most compounds populate new regions of chemical space. Compounds are also quite diverse, which is demonstrated in Figure 16, where the 50 in silico hits are organized in clusters. To quantify the degree of chemical novelty, we determined the maximum common substructure (MCS) (or largest common scaffold) between each in silico candidate and all compounds in the original RIPK1 dataset (“training” compounds), and we gathered the pairs of [hit; train] compounds that shared the largest scaffold.

The distribution of ratios of shared:non-shared atoms between each compound in the pair and their corresponding MCS is portrayed in Figure 15C. Both curves indicate that most hit-to-training pairs share about half (or less) of their structures, which implies that, in many cases, half (or more) of the structure in a hit compound is not observed in previous compounds with similar scaffolds. The shared MCSs are listed in Supporting Figure S12.

### 3.8. Phenotypic Necroptosis Inhibition Assay Reveals In Silico Hits with RIPK1 Inhibitory Activity

**Figure 17:**
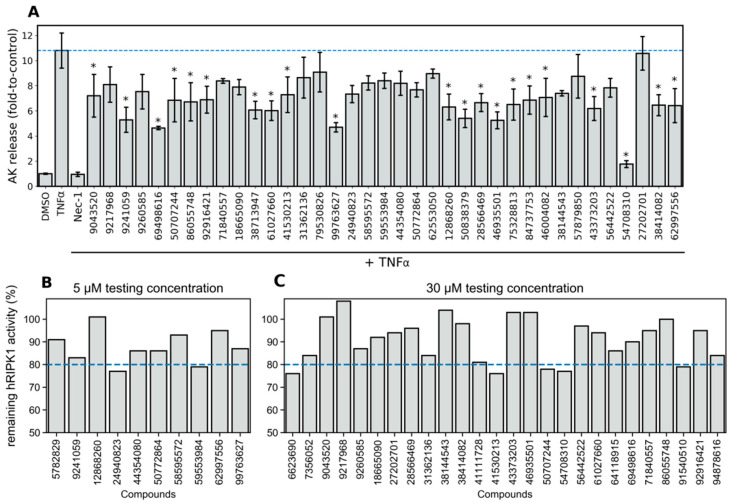
(**A**) Cell death measured by AK release produced under different treatment conditions in L929 cells, expressed as mean and standard deviation. The different cell treatment conditions are listed on the x-axis, which include a vehicle control (DMSO), a negative control (TNFα), a positive control (Nec-1), and the different test compounds paired with TNFα (N = 37). Of the initial 50 candidates, 3 were initially excluded due to solubility issues and 10 more were later excluded for not allowing reproducible AK release readouts (standard deviation greater than or equal to 30% of the mean), * *p* < 0.05 vs. TNFα. The remaining hRIPK1 activity was determined in a direct biochemical assay where a few compounds were initially shortlisted for testing at 5 µM (**B**), and the remaining compounds were later tested at 30 µM (**C**).

After the top 50 in silico hits were selected for purchase, we tested them experimentally as a small proof-of-concept for the usefulness of our in silico screening method. We first used a phenotypic assay of necroptosis inhibition, but unfortunately, three compounds (two of which were QSAR hits) indicated poor solubility and were excluded from experimental testing (Supporting File S1). In this assay, we induced cell death by incubating cells with TNFα for 8 h, and cell damage was evaluated through AK release. Co-incubation of cells with Nec-1 (control inhibitor) completely abolished TNFα-induced cell death, thus confirming necroptosis as the cell death mechanism. We compared the cell death response when treating cells with Nec-1 and the different in silico hits, and the results are indicated in Figure 17A. Notably, of the 47 compounds tested, 21 significantly inhibited TNFα-induced cell death (*p* < 0.05, Dunnett’s test). Furthermore, five of these compounds (9241059, 69498616, 99763627, 46935501, 54708310) were able to inhibit more than 50% of the cell damage caused by TNFα, while compound 54708310 indicated inhibition levels comparable to those of Nec-1 (1.76 and 0.94-fold AK release, respectively). The only QSAR-derived in silico hit that we were able to test (27202701) was among the inactives, which does not permit us to draw any conclusions on the performance of QSAR. Nonetheless, this is not surprising, seeing as its active prediction was associated with only 56% of the votes in the QSAR RF ensemble model, which indicates this was a challenging decision to make by the QSAR model, and likely results from the compound being close to the threshold that separates actives from inactives.

Next, we tested the compounds in a biochemical assay where their inhibitory activity was assessed directly against hRIPK1 in a cell-free assay. For this assay we only had a sufficient sample amount for 29 compounds and the results are portrayed in Figure 17. In an initial stage, we tested 10 compounds at a concentration of 5 μM (Figure 17B), and later, in a more conservative approach, we decided to test the remaining 19 compounds available at a concentration of 30 μM (Figure 17C). The results obtained from the direct hRIPK1 inhibition assay highlighted two compounds, 24940823 and 59553984, which indicated 20–25% hRIPK1 inhibition at 5 μM. This is a positive outcome since we selected these compounds from just 47 tested compounds which were shortlisted from a generic, diverse library using fast computational methods and little manual labor. Furthermore, the tested compounds represent new scaffolds, some of which are rather different than previously known actives. Indeed, the exploration of new chemical space (or “scaffold hopping”) is the biggest advantage of our approach, and these new compounds could presumably undergo the next step of hit optimization.

### 3.9. Agreement between the Phenotypic Assay of Necroptosis Inhibition in Murine L929 Cells and the Biochemical hRIPK1 Inhibition Assay

**Figure 18:**
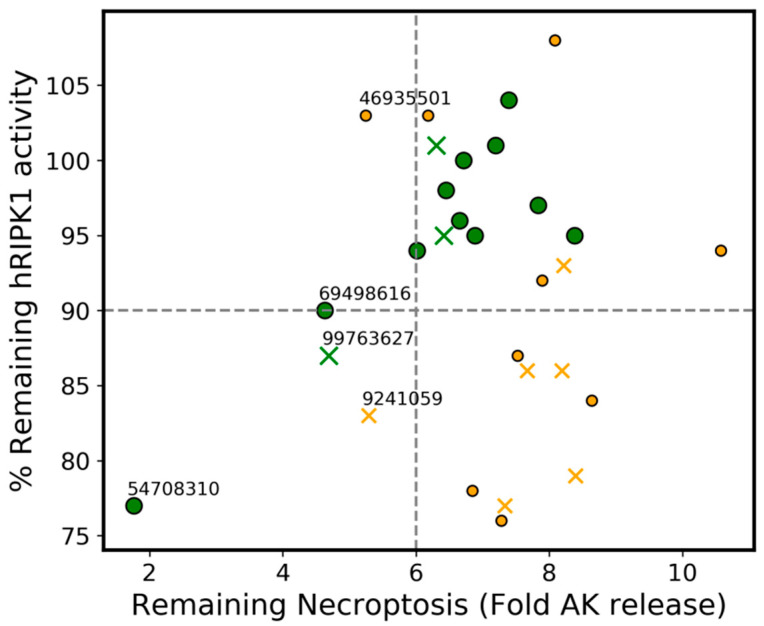
Qualitative correlation between phenotypic (X-axis) versus biochemical (Y-axis) readouts, with outliers detected by RANSAC identified in yellow. The “x” and “o” represent compounds tested at 5 μM and 30 μM, respectively, in the biochemical (hRIPK1 activity) assay. The labelled compounds are the top 5 candidates from the phenotypic assay. The grey dashed lines are a simple aid for qualitative analysis.

Mouse and human necroptosis are not expected to correlate well due to flexibility constraints of the allosteric pocket in the mouse RIPK1 [13], so we wanted to (retrospectively) check how predictive, in qualitative terms, the phenotypic screen (L929 cells) would be in shortlisting RIPK1 actives. To achieve this, we analyzed the data through the outlier detection algorithm RANSAC [43] (7% *residual_threshold*, 1000 *max_trials*, *stop_probability* of 0.90) using the 30 μM assay data only. Even when using a much more restrictive residual threshold, the RANSAC algorithm spontaneously detected the robust trend in green (Figure 18). However, RANSAC was used merely as a visual aid since it is not reasonable to expect a linear correlation between mouse and human readouts. We concluded that, overall, high necroptosis inhibitory values (i.e., low-fold AK release) are associated with relatively low hRIPK1 inhibition. This is observed with compound 54708310, which has anti-necroptosis activity comparable to that of Nec-1, but only indicates 77% hRIPK1 inhibition. Additionally, the phenotypic assay indicated four compounds (59553984, 41530213, 50707244 and 24940823) as having a very low inhibitory activity (orders of magnitude lower than 54708310). However, these compounds indicate similar (if not lower) hRIPK1 inhibition to that of 54708310.

The four disproportionately more inactive compounds produced from the phenotypic necroptosis assay could correspond to mRIPK1-inactives, which are hRIPK1-actives (the difference arising from the lack of flexibility in the mRIPK1 pocket). Alternatively, it is possible that these compounds have limited permeation through the cell membrane. With respect to the one compound which is disproportionately more active in the phenotypic assay than in the biochemical assay (54708310), this compound may target another protein in the necroptosis cascade which is supported by several dual RIPK1-RIPK3 inhibitors [44], as these proteins share high sequence similarity (32.3% sequence identity, considering high similarity is typically observed for values above 30% [45]). For example, the target prediction model built by ChEMBL using conformal QSAR [18] predicts this compound targets PARP1 (with 90% confidence), which is a protein that is an immediate signaling network neighbor of RIPK1 (according to OmniPath’s curated network of September 2020 [46]). Finally, one cannot discard the possibility of compound degradation during shipment to the location where the RIPK1 inhibition assay was performed, which could cause differences between both assays.

### 3.10. Extracting General Structure-Activity Relationships (SAR) in New RIPK1 Inhibitors

All 50 in silico candidates spanned into the “foot” portion of the boot-shaped allosteric back pocket (foot starts at 6.47 Å from the far end of the pocket), although the docking sphere used during docking calculations allowed binding to a portion of the ATP-binding site (See Figure 2). In fact, 52% of ligands were placed at 3 Å or less from the far end of the pocket, resembling the placement observed for most co-crystal ligands in the PDB structures available to date. There was, however, no correlation between minimum distance to the bottom of the pocket and activity (some of the most inactive compounds were also placed at less than 3 Å of the end of the pocket). Ultimately this implies that the distance to the end of the pocket cannot be used as a standalone parameter to predict activity. Like the co-crystal ligands, our in silico candidates also indicate a binding primarily driven by hydrophobic contacts. A table with all residue contacts is available in Supporting Figures S13–S15.

**Figure 19:**
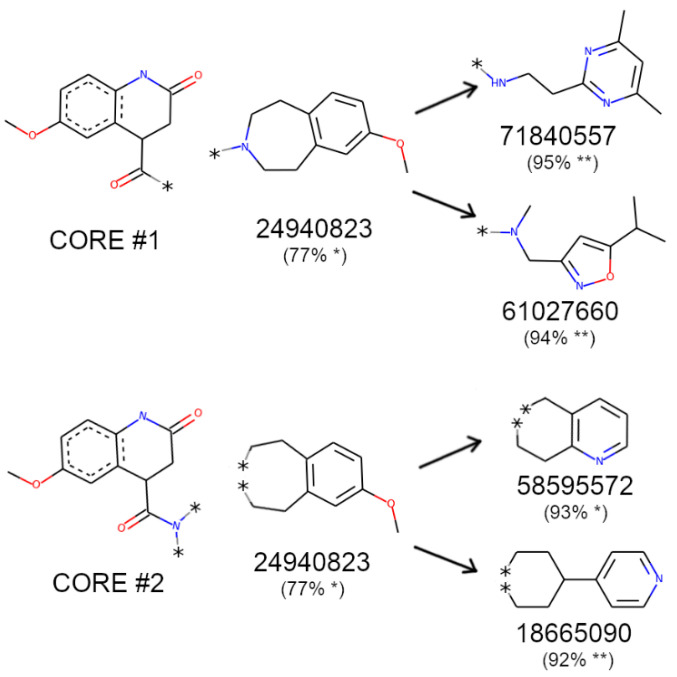
R-group decomposition for the most active compounds found among in silico candidates with activities (% remaining hRIPK1 activity) portrayed in parenthesis. * tested at 5 μM concentration; ** tested at 30 µM concentration.

To extract meaningful structure-activity relationships, we performed an R-group decomposition analysis using RDKit, focusing on the most active candidates, i.e., compounds with RIPK1 inhibition ≥ 20% (N = 7, listed in Supporting Figure S16). After an exhaustive search of all possible R-group modifications sharing the same core structure, we discovered several examples of active–inactive pairs sharing the same core (Supporting Figures S17–S19). Interestingly, for almost all pairs, the core was located at the closed end of the pocket which implies the modifications associated with a shift in activity occurred at the entrance of the pocket. With respect to the most active RIPK1 inhibitor compound (24940823) found from the biochemical assay, it shared a common core with four other inactive compounds (Figure 19).

**Figure 20:**
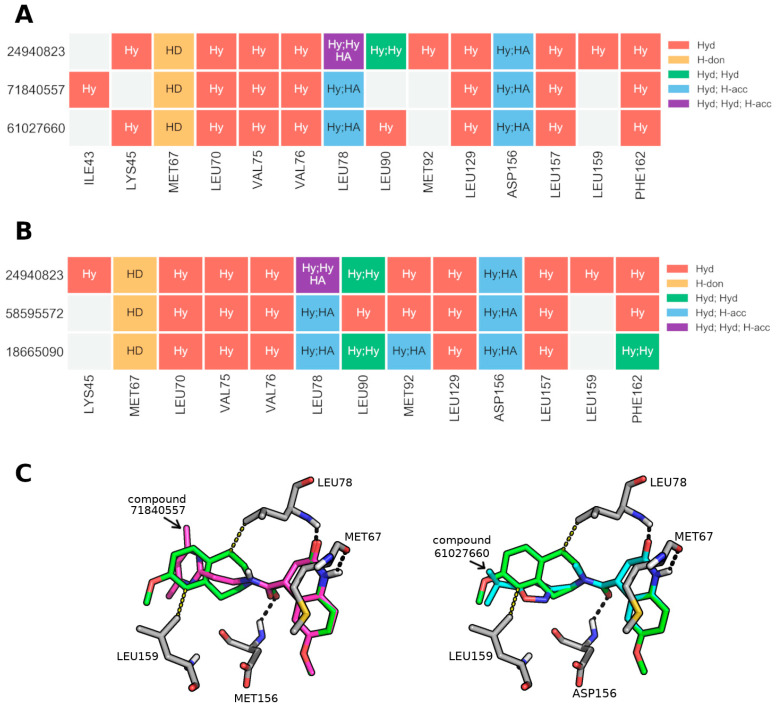
Protein–ligand interactions for the pairs in the SAR core #1 (**A**) and core #2 (**B**) involving the most active compound (24940823) in the RIPK1 inhibition assay. Hyd: hydrophobic contact, H-don: H-donor, H-acc: H-acceptor. (**C**) Docked poses of the compounds (71840557 in magenta and 61027660 in cyan) sharing the first core with 24940823 (in green). Only key differing contacts are proven to prevent the figure from becoming overly crowded with residues.

For the first pair of compounds sharing an R group with the most active inhibitor, the shared core was perfectly aligned between all three, and they differed only in the placement of portions closest to the pocket entry. Despite this difference, all molecules shared a very similar residue interaction profile, but some key changes were identified: (1) the existence of an extra hydrophobic contact between the most active compound 24940823 and LEU78, which is a residue located halfway across the length of the pocket, and (2) a hydrophobic contact with LEU159, which is near the entrance of the pocket (both contacts portrayed in Figure 20). It is possible that these additional contacts helped stabilize the complex and therefore explain the increased activity. This is particularly relevant since LEU78 belongs to the binding loop and is located above and to the left of the pocket, while LEU159 belongs to the activation loop which is located to the right of the pocket (when looking straight through the entrance of the pocket). The loss of both contacts can be ascribed to the large lipophilic moiety (two fused rings) being replaced with a single ring with multiple polar atoms, connected to the core by an aliphatic linker (see Figure 19). In fact, in both inactive compounds, the added aromatic N or O atoms are placed in a largely lipophilic part of the pocket, near LEU159’s lipophilic side chain.

Interestingly, comparing the binding pose of compound 24940823 with the co-crystal ligand of 5HX6 indicates that the former turns away from the entrance toward the ATP-binding site (see Supporting Figure S20 and Figure 2).

## 4. Conclusions

Necroptosis inhibition has been intensely explored in the last decades to develop therapies for inflammatory and neurodegenerative diseases and acute ischemic injuries. RIPK1, a major biological player in the necroptotic cascade, is a very appealing target due to an unusual motif within its binding pocket that can be leveraged to produce inhibitors with excellent selectivity. Because of the attention given to this protein, there are now hundreds of compounds in the low micromolar and nanomolar range. However, unlike other kinases, no effort has yet been made to take advantage of these data to better understand RIPK1 inhibitors and to create general in silico predictive models that can be used to find new compounds. To bridge this gap, we propose here the first validated in silico model for the prediction of RIPK1 inhibitors. The model devised is particularly geared towards finding new potentially interesting scaffolds that can be used as starting points in the development of RIPK1 inhibitors.

In this work we have built two alternative in silico models. The first was derived from molecular docking and consisted of a four-residue (LEU70, VAL75, ASP156, LEU157) signature filter and a six-residue (LEU70, VAL75, LEU78, LEU129, ASP156, ASP157) similarity filter used in consensus and built from available ChEMBL data. Both models have good predictive performance (>90 and 92% precision, respectively) when used to find actives among a set of compounds, and this performance has been validated for ChEMBL data in addition to compounds from two other datasets (the Harris and Roche datasets). These serve as an alternative to docking scores. This is an important aspect, as docking scores are typically used to predict new actives but here indicated a poor screening power, as it was not able to differentiate actives from inactives among ChEMBL compounds. These results highlight the importance of thoroughly validating a docking model because, in our case, even when good self- and cross-docking are obtained, the model can still perform poorly on the task of actually shortlisting new hits. Additionally, the four-residue signature aligns with empirical evidence of the importance of each of these residues in the structural integrity and function of RIPK1. Both interaction-based models derived from docking offer a simple and intuitive approach to screen for new actives but, nevertheless, users should be mindful that both models have their own applicability domains and might not behave as well in other regions of chemical space that were not explored in this work.

The second in silico model corresponded to a machine learning QSAR model from data collated from ChEMBL, literature, patents, and PubChem data. This model also allowed for good prediction of actives (96.4% precision). The model was trained with 200 RDKit molecular descriptors and the six key residue contacts, using a Random Forest algorithm and the error rate for accepted predictions was controlled using an applicability domain filter. Although there are only six residues that competed with a much larger set of physicochemical descriptors, they all were spontaneously selected with relatively high feature importance.

Notably, when screening the ChemBridge database of purchasable compounds, this model selected only a few dozen hits among several thousand compounds screened, but all of the hits overlapped those from the two molecular docking-derived models (PLIFs similarity and 4-contact signature). A set of 50 proof-of-concept compounds were selected from the ChemBridge database for experimental testing, all of which occupied new regions of chemical space. This is evidenced by the fact that they shared a relatively small common substructure with previously tested inhibitors. This is particularly useful as it promotes innovation in the drug discovery of RIPK1 inhibitors.

The proof-of-concept compounds were selected using a consensus of both methods and tested in both a phenotypic necroptosis inhibition assay and in a biochemical RIPK1 inhibition assay. Although no potent actives were found, 7 of the 47 tested compounds indicated about 20–25% RIPK1 inhibition but, more importantly, were found to occupy relatively new areas of chemical space. Considering that a very large in vitro high-throughput screening campaign may only produce weakly active compounds as well, we believe our screening method may be useful as it allows for exploring completely new areas of chemical space in a very fast and inexpensive manner, and has been proven to produce various starting points for further hit and hit-to-lead optimization campaigns.

## Data Availability

The PDB files of RIPK1 used were obtained from the Protein Data Bank (PDB) at www.rscb.org (accessed on 2 March 2019). Bioactivity data was sourced from ChEMBL using the sqlite database (downloaded from https://ftp.ebi.ac.uk/pub/databases/chembl/ChEMBLdb/latest/ (accessed on 20 February 2019)), from PubChem BioAssays in pubchem.ncbi.nlm.nih.gov (accessed on 20 February 2019), and from a patent and a publication both in the public domain. MOE2019.0901, used for protein and compound preparation, is available upon license purchasing, and the open-access version of PyMOL was used to prepare images of 3D structures. GOLD, used for docking calculations, is available upon license purchasing. The PLIFs-detection python library, plip v.1.4.4, was installed from the GitHub repository (https://github.com/pharmai/plip (accessed on 2 March 2019)). OPSIN and OSRA are free to use as web tools (at https://opsin.ch.cam.ac.uk/ (accessed on 2 March 2019) and https://cactus.nci.nih.gov/cgi-bin/osra/index.cgi (accessed on 2 March 2019)). PLIP-tools Data handling and analysis were done in Jupyter with Python 3.7 and all other python modules mentioned are also free to use (installed through conda or pip). The final dataset can be reproduced following the steps in the methods and scripts will be made available upon request.

## Supplementary Materials

The following supporting information can be downloaded at: https://www.mdpi.com/article/10.3390/molecules27154718/s1, Figure S1: Distribution of activities in the final RIPK1 inhibitors dataset (excluding PubChem Inactives as their activity value is qualitative in nature). Figure S2. (A) Number of cross-docked ligands (out of 8) with a top-scored docking pose within 2 Å of their experimental pose. The top-scored pose is obtained from a total of 1000 poses. (B) RMSD between self- or cross-docked poses and the corresponding co-crystal ligand, using GoldScore. Figure S3. Robustness of poses found when using 5HX6 versus 5TX5. Docking calculations with 5TX5 produced an extremely high RMSD of solutions found for 4ITI-lig when varying the number of poses used (50 vs. 1000). This is an indication that using 5TX5 might generate non-robust solutions for any given ligand. Figure S4. Comparing the docking scores (GoldScore) obtained from using 1000 vs. 50 on the ChEMBL dataset, to establish whether it is feasible to use 50 poses for the larger screening step. Table S1. Characteristics of the binding pocket composed of the allosteric binding site and the ATP-binding site (pockets are automatically detected by DoGSiteScorer in ProteinPlus and as both binding sites are adjacent they cannot be perfectly separated). The smaller “ellipsoid b/a” is, the more elongated the pocket is; the smaller “ellipsoid c/a” is the more narrow the pocket is. Figure S5. (A) Ranking of actives and inactives from the three datasets added after ChEMBL (Harris dataset, Roche dataset and PubChem Inactives). (B) Enrichment curves for ChEMBL data as a function of the ligand efficiency limit, expressed as a percentile, for 5HX6 structure (50 poses). The enrichment using 4ITH’s ligand efficiencies is also shown to cover the possibility that ChEMBL molecules perform better in this alternative pocket. Increasing the minimum ligand efficiency score at which predictions are accepted leads to no meaningful enrichment of covered actives over inactives. Figure S6. Exhaustive analysis of all possible combinations of the 6 key residues (LEU70, VAL75, LEU78, LEU129, ASP156, ASP157) and their corresponding difference (delta) of the percentage of actives versus inactives covered by that combination. The values labelled at the top of the plot indicate the % actives that show the corresponding signature, and the * indicates the combination od residues with the largest delta. blue: combinations of 2 residues; green: combinations of 3 residues; red: combinations of 4 residues; yellow: combinations of 5 residues; purple: a combination of all 6 residues. Figure S7. Difference perspectives of the 6 shared residues among the 8 X-ray structures covered by this work and their location around RIPK1’s allosteric back pocket. The residues that make up the 4-residue signature are shown in grey. Supporting Figure S8. Distribution of values in ChemBridge. Figure S9. Purchased in silico hits overlapping between docking and QSAR. Figures S10 and S11. Purchased in silico hits obtained from docking. Figure S12. shared maximum common substructures between new in silico hits and previously known compounds in the RIPK1 dataset. Figure S13. PLIFs for the co-crystal ligand of RIPK1. Figure S14. PLIFs for the compounds with remaining hRIPK1 activity < 80%. Figure S15. PLIFs for the remaining compounds beyond those with remaining hRIPK1 activity > 80%. Figure S16. Co-crystal ligand (cyan) and each of the most promising in silico candidates (remaining hRIPK1 activity <80%) docked in the allosteric back pocket of RIPK1 (PDB:5HX6). Figure S17. SARs involving compound 24940823. Both cores identified for Structure-Activity pairs are turned to the end of the allosteric back pocket in RIPK1 (see Figure S15); Figure S18. SARs involving compound 59553984. The core identified for the Structure-Activity pair is turned to the end of the allosteric back pocket in RIPK1 (see Figure S15); Figure S19. SARs involving compound 50707244. The core identified for Structure-Activity pair is turned to the end of the allosteric back pocket in RIPK1 (see Figure S15); Figure S20. Co-crystal ligand (cyan) and most active in silico hit 24920823 (green) docked in the allosteric back pocket of RIPK1 (PDB:5HX6).
