# Supplementary material for: Harnessing Protein-Ligand Interaction Fingerprints to Predict New Scaffolds of RIPK1 Inhibitors
Source: Molecules. 2022 Jul 23;27(15):4718. doi: 10.3390/molecules27154718 (PMC9330098; doi:10.3390/molecules27154718)
Supplement: Supplementary file 1 [file molecules-27-04718-s001.zip › molecules-1813194-supplementary.pdf]

Supporting Information

Harnessing protein-ligand interaction fingerprints to predict new scaffolds of RIPK1 inhibitors

Natália Aniceto, Vanda Marques, Joana Amaral, Patrícia Serra, Rui Moreira, Cecília M. P. Rodrigues, Rita C. Guedes*

Department of Pharmaceutical Sciences and Medicines and Research Institute for Medicines (iMed.ULisboa), Faculdade de Farmácia, Universidade de Lisboa, 1649-003 Lisboa, Portugal

*Corresponding author. email: rguedes@ff.ulisboa.pt

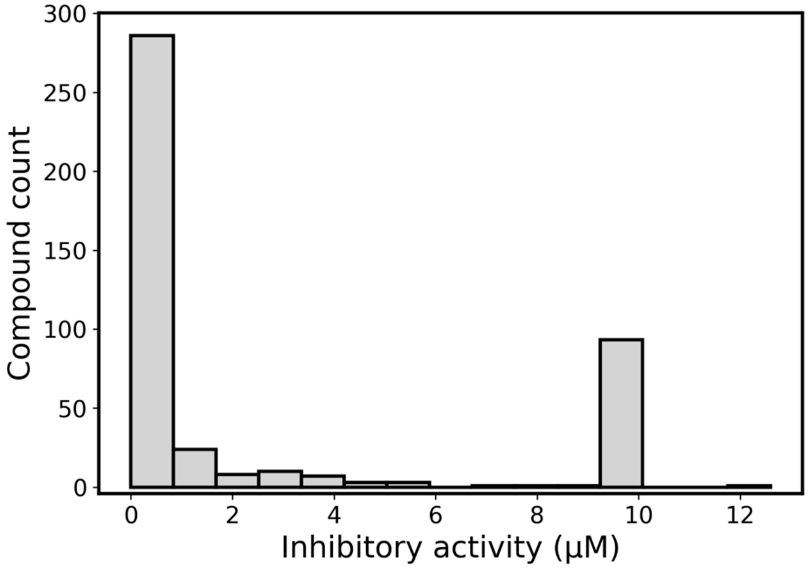

Supporting Figure S1. Distribution of activities in the final RIPK1 inhibitors dataset (excluding PubChem Inactives as their activity value is qualitative in nature).

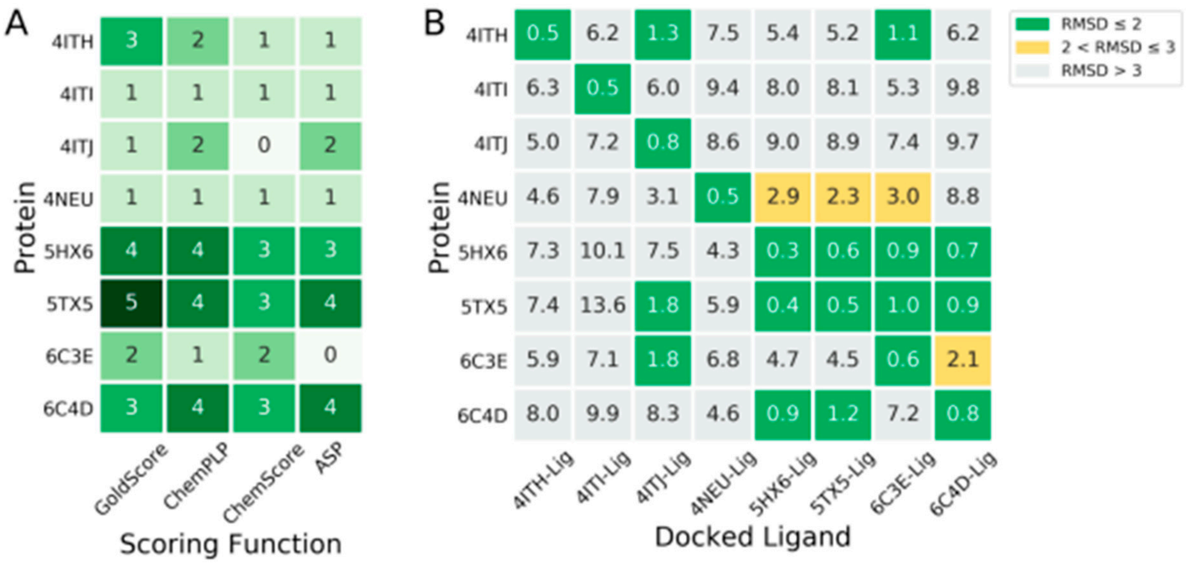

Supporting Figure S2. (A) Number of cross-docked ligands (out of 8) with a top-scored docking pose within 2 Å of their experimental pose. The top-scored pose is obtained from a total of 1000 poses. **(B)** RMSD between self- or cross-docked poses and the corresponding co-crystal ligand, using GoldScore.

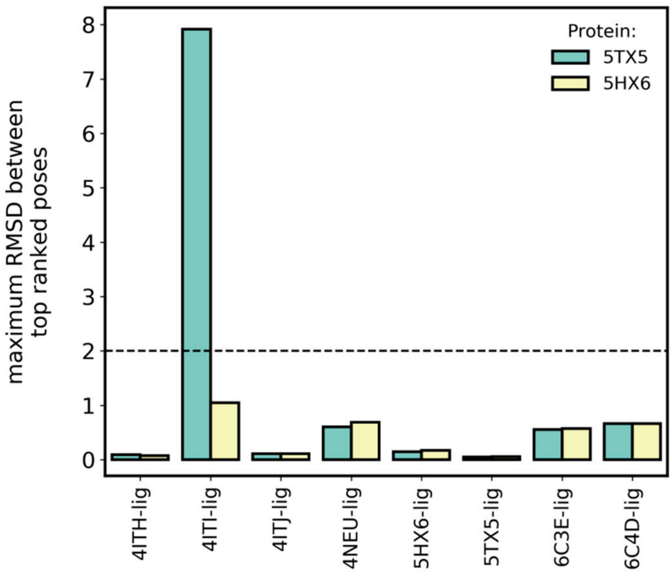

Supporting Figure S3. Robustness of poses found when using 5HX6 versus 5TX5. Docking calculations with 5TX5 produced an extremely high RMSD of solutions found for 4ITI-lig when varying the number of poses used (50 vs 1000). This is an indication that using 5TX5 might generate non-robust solutions for any given ligand.

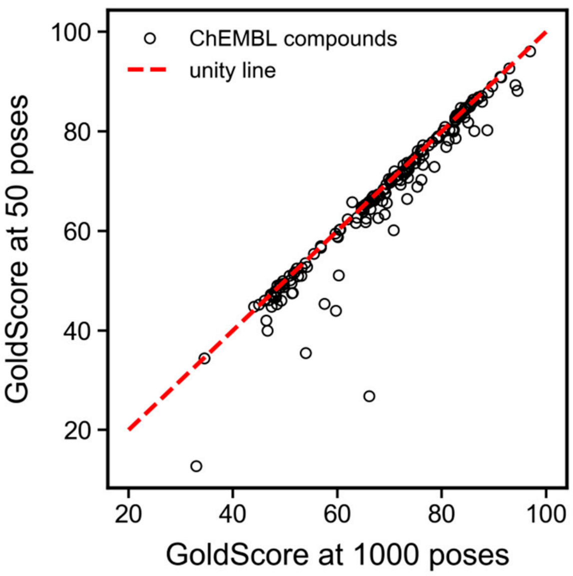

Supporting Figure S4. Comparing the docking scores (GoldScore) obtained from using 1000 vs 50 on the ChEMBL dataset, to establish whether it is feasible to use 50 poses for the larger screening step.

Supporting Table S1. Characteristics of the binding pocket composed of the allosteric binding site and the ATP-binding site (pockets are automatically detected by DoGSiteScorer in ProteinPlus and as both binding sites are adjacent they cannot be perfectly separated). The smaller “ellipsoid b/a” is, the more elongated the pocket is; the smaller “ellipsoid c/a” is the more narrow the pocket is.

						narrowness of the pocket	*how elongated is the pocket?*
**Name**	**Volume (Å³)**	**Surface (Å²)**	**Drug Score**	**Simple Score**	**depth (Å)**	**ellipsoid c/a**	**ellipsoid b/a**
4ITH	392.9	352.42	0.73	0.24	16.44	0.26	0.43
4ITI	786.62	823.15	0.84	0.52	19.9	0.45	0.51
4ITJ	702.21	786.99	0.83	0.45	19.26	0.2	0.32
4NEU	905.79	1017.21	0.82	0.82	21.24	0.08	0.28
5HX6	876.1	1041.65	0.82	0.8	20.72	0.17	0.24
5TX5	545.28	591.91	0.76	0.57	19.17	0.1	0.23

6C3E	439.68	337.79	0.65	0.45	18.96	0.14	0.25
6C4D	711.62	763.75	0.79	0.7	16.95	0.15	0.23

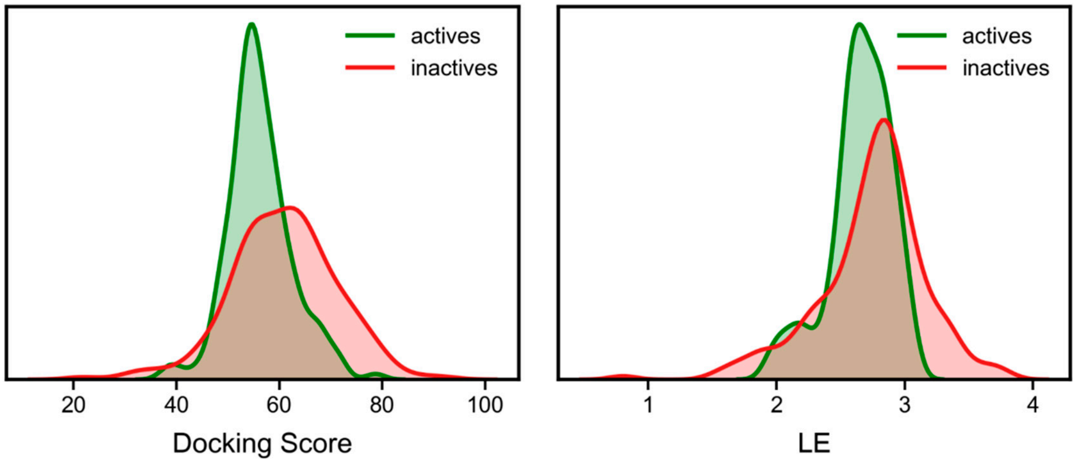

(**A**)

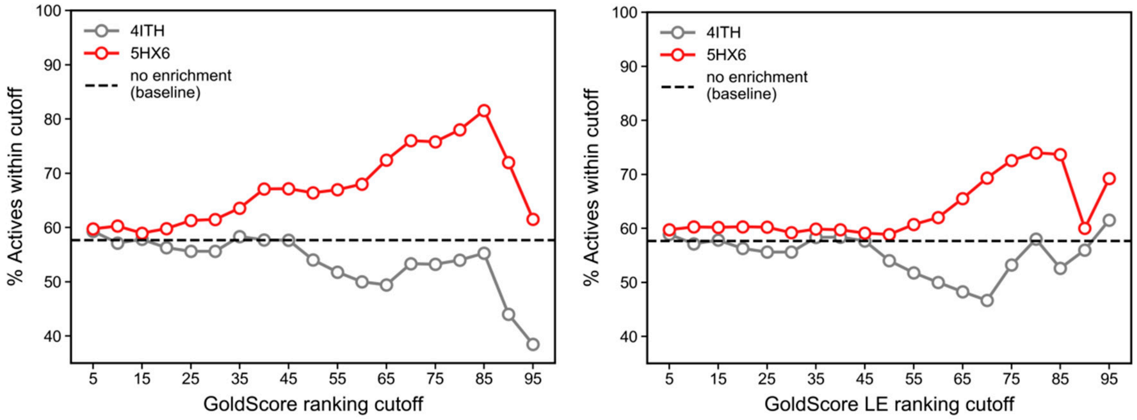

(**B**)

Supporting Figure S5. (A) Ranking of actives and inactives from the three datasets added after ChEMBL (*Harris dataset, Roche dataset and PubChem Inactives).* **(B)** Enrichment curves for ChEMBL data as a function of the ligand efficiency limit, expressed as a percentile, for 5HX6 structure (50 poses). The enrichment using 4ITH's ligand efficiencies is also shown to cover the possibility that ChEMBL molecules perform better in this alternative pocket. Increasing the minimum ligand efficiency score at which predictions are accepted leads to no meaningful enrichment of covered actives over inactives.

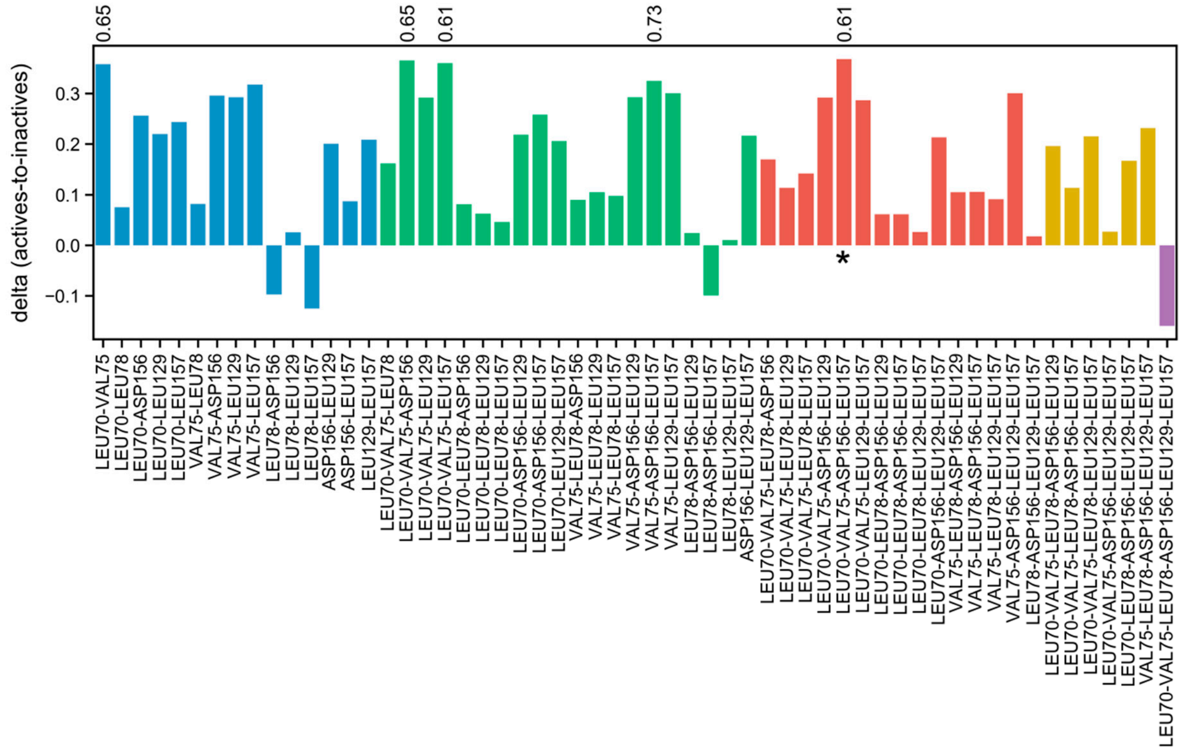

Supporting Figure S6. Exhaustive analysis of all possible combinations of the 6 key residues (LEU70, VAL75, LEU78, LEU129, ASP156, ASP157) and their corresponding difference (delta) of percentage of actives versus inactives covered by that combination. The values labelled at the top of the plot indicate the % actives that show the corresponding signature, and the * indicates the combination od residues with the largest delta. blue: combinations of 2 residues; green: combinations of 3 residues; red: combinations of 4 residues; yellow: combinations of 5 residues; purple: combination of all 6 residues.

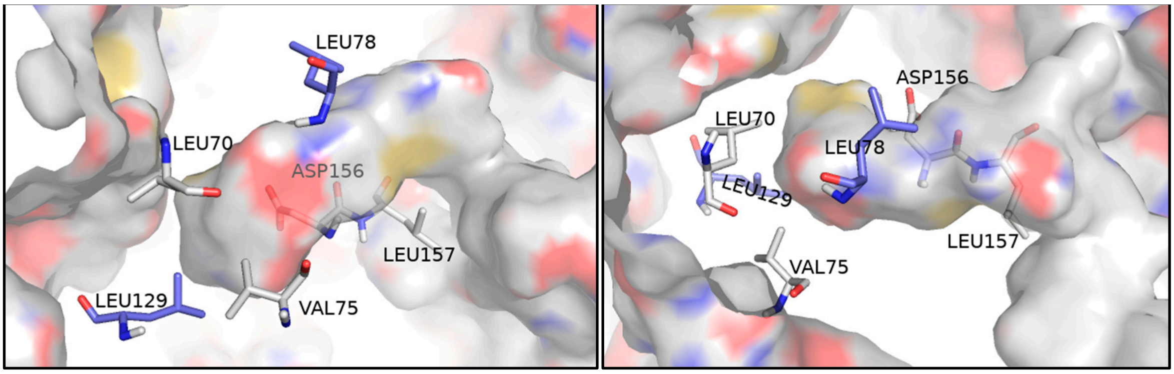

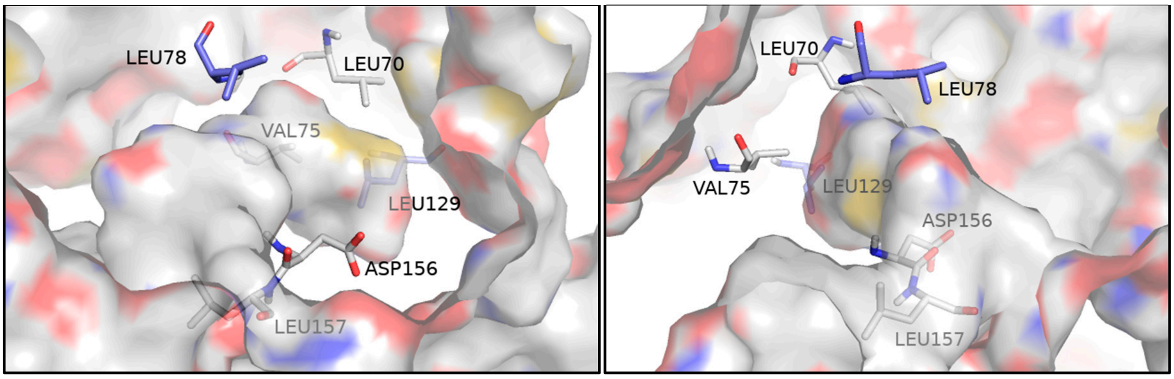

Supporting Figure S7. Difference perspectives of the 6 shared residues among the 8 X-ray structures covered by this work and their location around RIPK1's allosteric back pocket. The residues that make up the 4-residue signature are shown in grey.

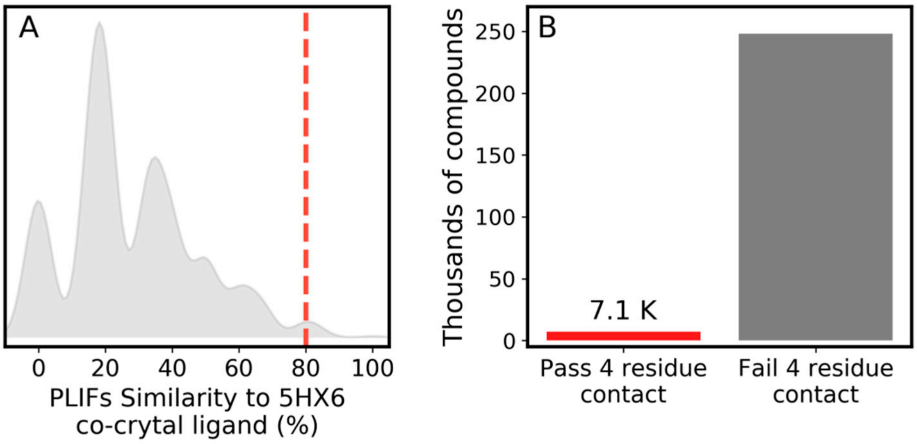

Supporting Figure S8. Distribution of values in ChemBridge (**A**,**B**).

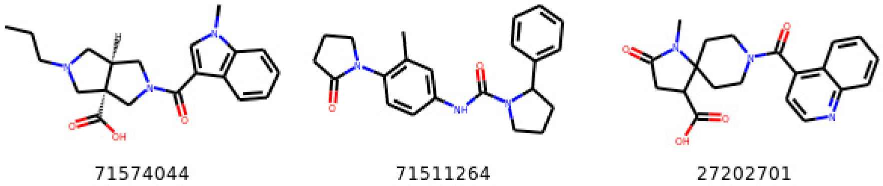

Supporting Figure S9. Purchased in silico hits overlapping between docking and QSAR.

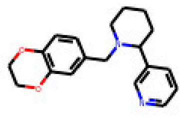

5782829

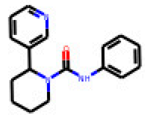

6342385

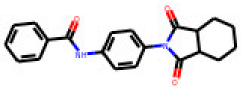

6623690

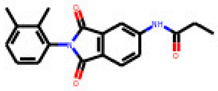

7356052

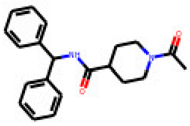

9043520

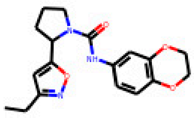

9217968

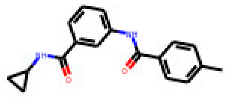

9241059

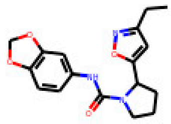

9260585

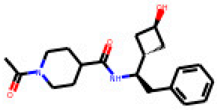

64118915

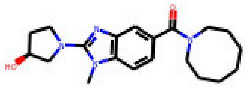

69498616

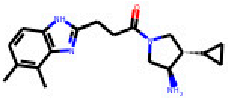

41111728

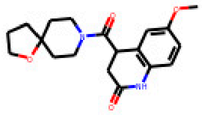

50707244

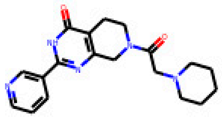

25795351

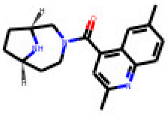

86055748

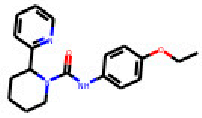

92916421

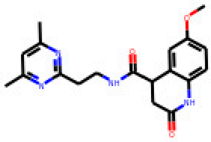

71840557

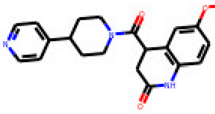

18665090

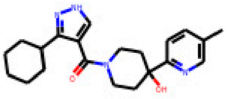

38713947

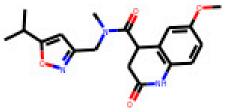

61027660

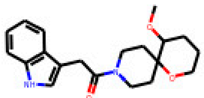

41530213

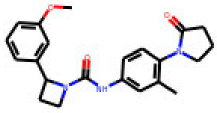

91540510

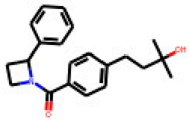

94878616

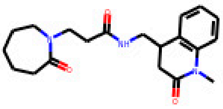

60149832

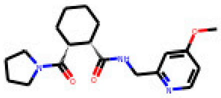

31362136

Supporting Figure S10. Purchased in silico hits obtained from docking (part 1).

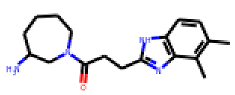

79530826

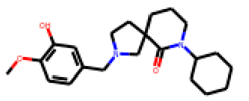

99763627

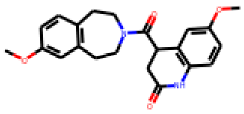

24940823

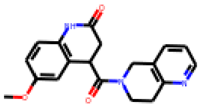

58595572

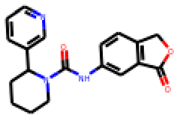

59553984

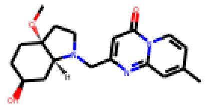

44354080

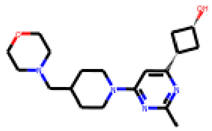

50772864

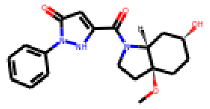

62553050

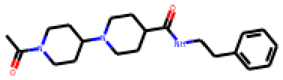

12868260

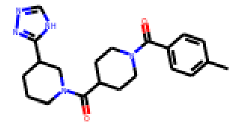

50838379

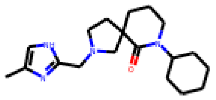

28566469

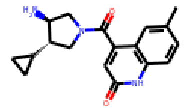

46935501

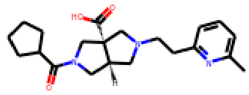

75328813

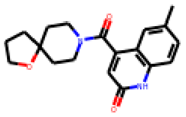

84737753

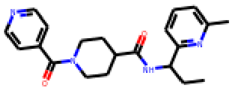

46004082

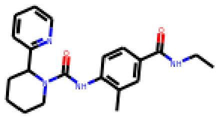

38144543

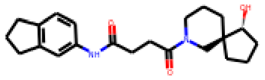

57879850

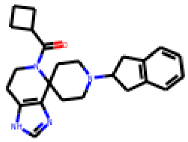

43373203

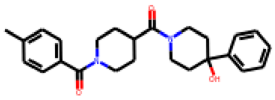

56442522

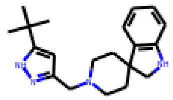

54708310

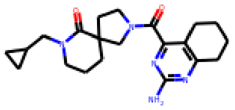

38414082

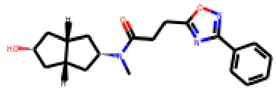

62997556

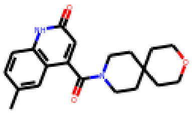

35086385

Supporting Figure S11. Purchased in silico hits obtained from docking (part 2).

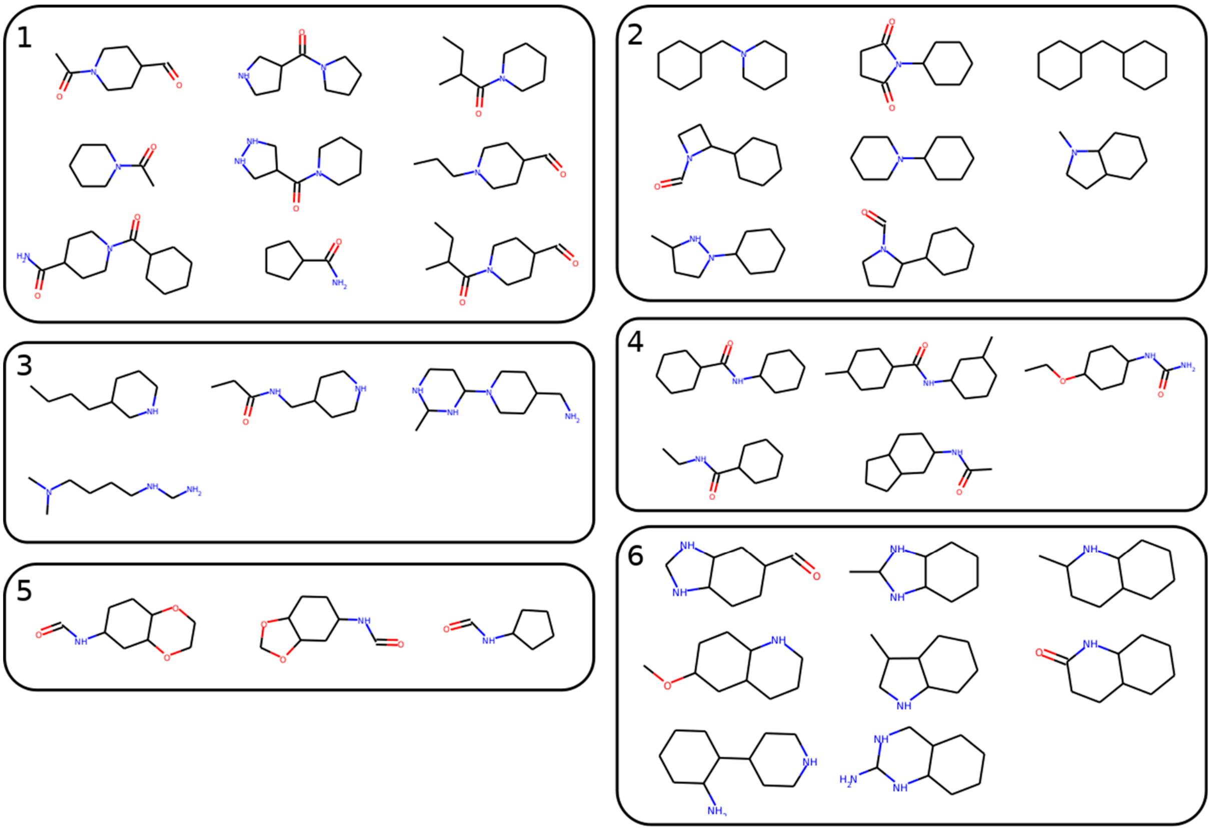

Supporting Figure S12. shared maximum common substructures between new in silico hits and previously known compounds in the RIPK1 dataset.

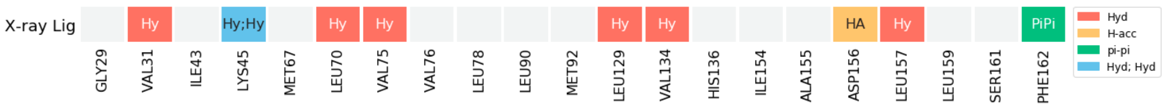

Supporting Figure S13. PLIFs for the co-crystal ligand of RIPK1.

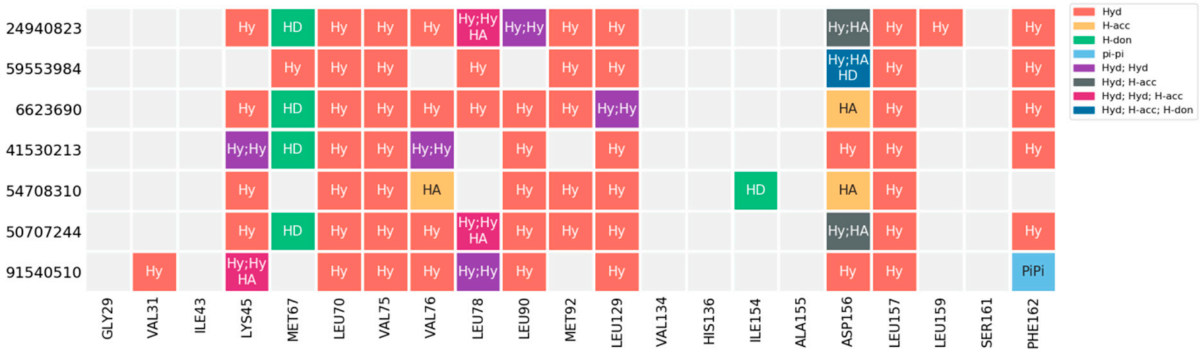

Supporting Figure S14. PLIFs for the compounds with remaining hRIPK1 activity < 80%.

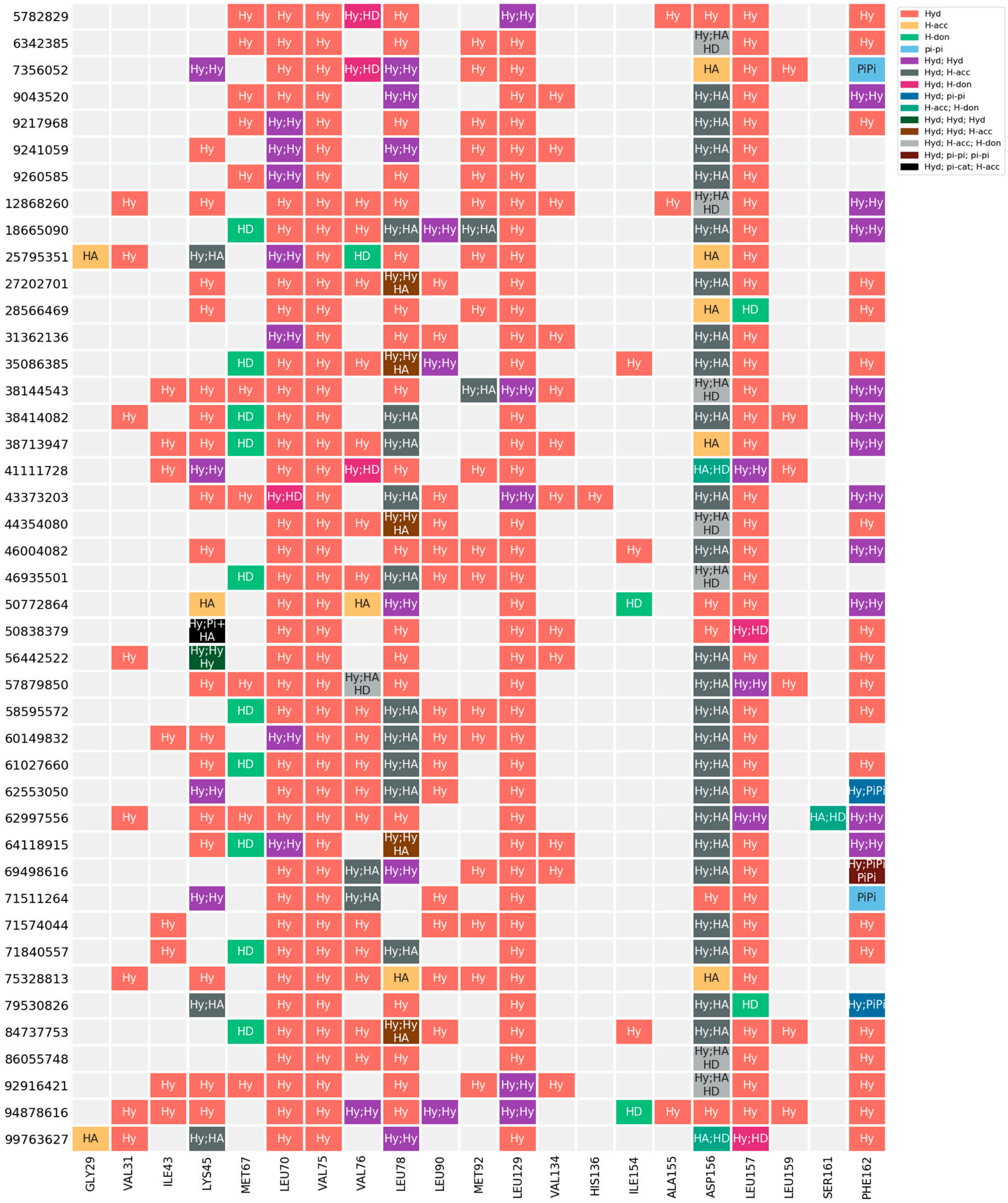

Supporting Figure S15. PLIFs for the remaining compounds beyond those with remaining hRIPK1 activity > 80% .

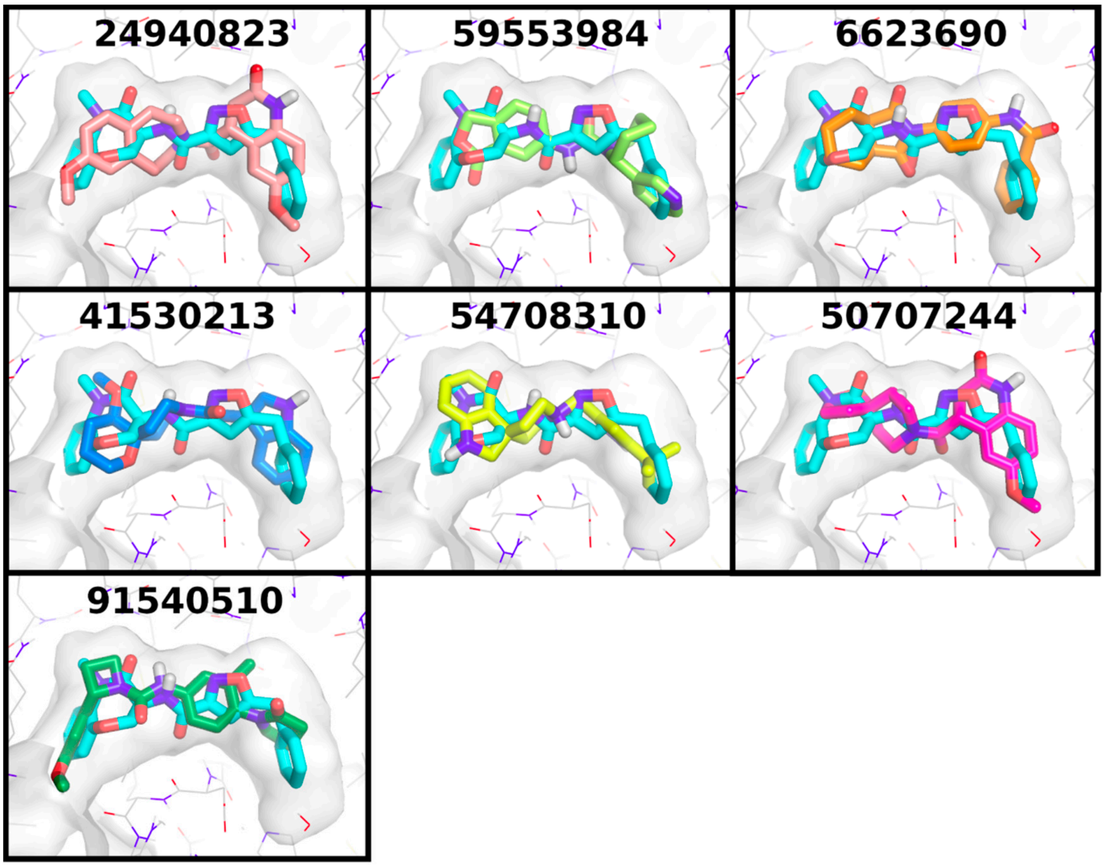

Supporting Figure S16. Co-crystal ligand (cyan) and each of the most promising *in silico* candidates (remaining hRIPK1 activity <80%) docked in the allosteric back pocket of RIPK1 (PDB:5HX6).

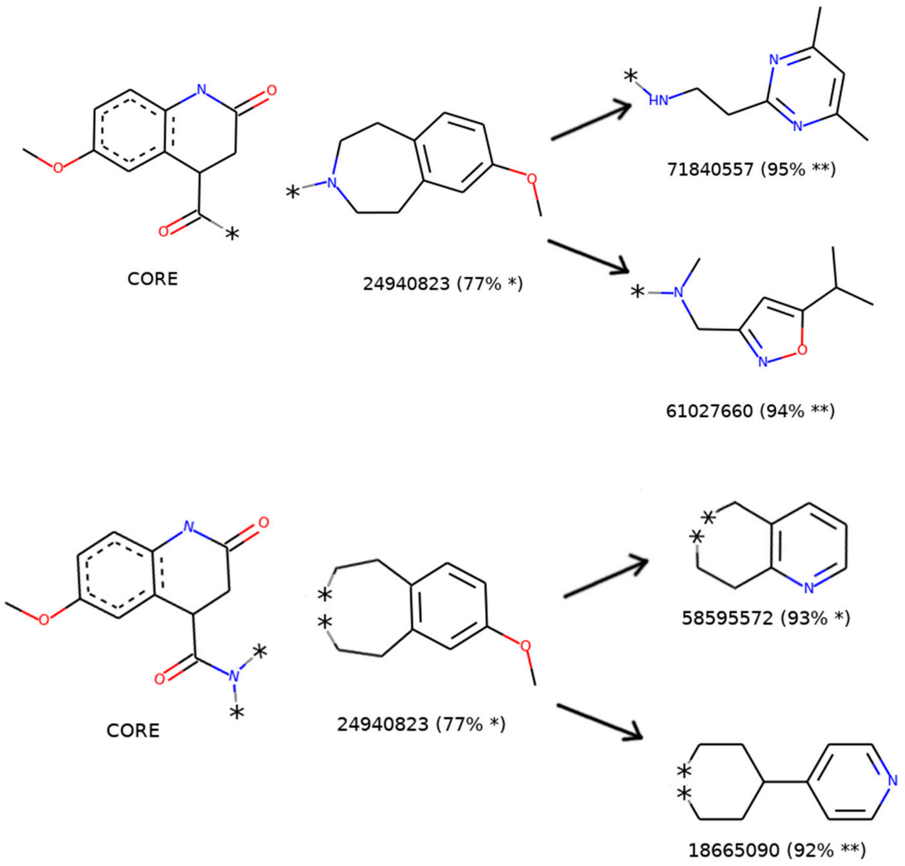

Supporting Figure S17. SARs involving compound 24940823. Both cores identified for Structure-Activity pairs are turned to the end of the allosteric back pocket in RIPK1 (see **Figure S15)**

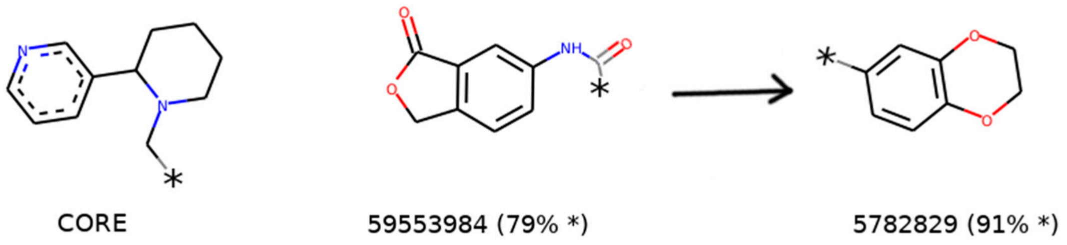

Supporting Figure S18. SARs involving compound 59553984. The core identified for Structure-Activity pair is turned to the end of the allosteric back pocket in RIPK1 (see **Figure S15)**

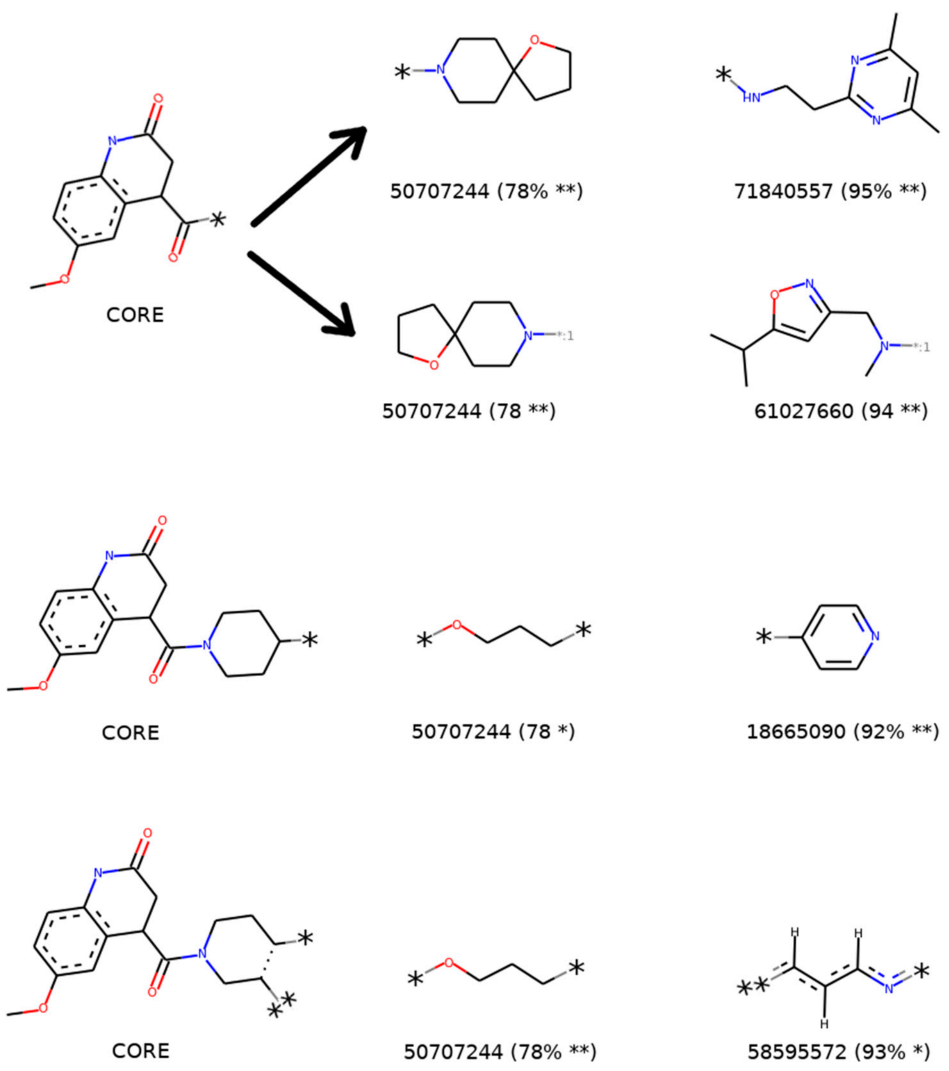

Supporting Figure S19. SARs involving compound 50707244. The core identified for Structure-Activity pair is turned to the end of the allosteric back pocket in RIPK1 (see **Figure S15)**

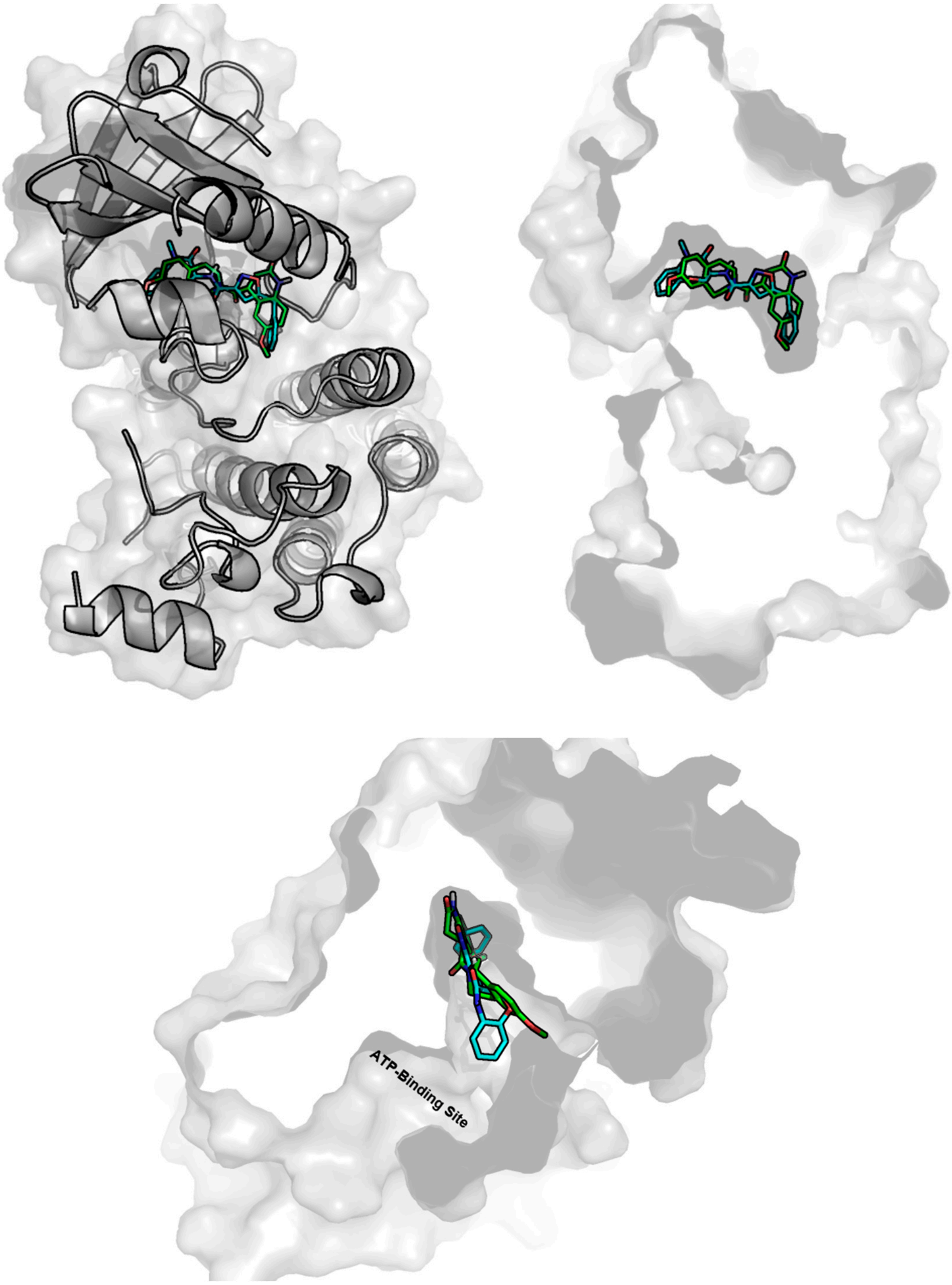

Supporting Figure S20. Co-crystal ligand (cyan) and most active in silico hit 24920823 (green) docked in the allosteric back pocket of RIPK1 (PDB:5HX6).
